# Molecular architecture of the Chikungunya virus replication complex

**DOI:** 10.1126/sciadv.add2536

**Published:** 2022-11-30

**Authors:** Yaw Bia Tan, David Chmielewski, Michelle Cheok Yien Law, Kuo Zhang, Yu He, Muyuan Chen, Jing Jin, Dahai Luo

**Affiliations:** ^1^ Lee Kong Chian School of Medicine, Nanyang Technological University, EMB 03-07, 59 Nanyang Drive, Singapore 636921, Singapore.; ^2^ NTU Institute of Structural Biology, Nanyang Technological University, EMB 06-01, 59 Nanyang Drive, Singapore 636921, Singapore.; ^3^ Biophysics Graduate Program, Departments of Bioengineering, and of Microbiology and Immunology, Stanford University, Stanford, CA 94305, USA.; ^4^ Division of CryoEM and Bioimaging, SSRL, SLAC National Accelerator Laboratory, Stanford University, Menlo Park, CA 94025, USA.; ^5^ Vitalant Research Institute, San Francisco, CA 94118, USA.; ^6^ Department of Laboratory Medicine, University of California, San Francisco, San Francisco, CA 94143, USA.

## Abstract

To better understand how positive-strand (+) RNA viruses assemble membrane-associated replication complexes (RCs) to synthesize, process, and transport viral RNA in virus-infected cells, we determined both the high-resolution structure of the core RNA replicase of chikungunya virus and the native RC architecture in its cellular context at subnanometer resolution, using in vitro reconstitution and in situ electron cryotomography, respectively. Within the core RNA replicase, the viral polymerase nsP4, which is in complex with nsP2 helicase-protease, sits in the central pore of the membrane-anchored nsP1 RNA-capping ring. The addition of a large cytoplasmic ring next to the C terminus of nsP1 forms the holo-RNA-RC as observed at the neck of spherules formed in virus-infected cells. These results represent a major conceptual advance in elucidating the molecular mechanisms of RNA virus replication and the principles underlying the molecular architecture of RCs, likely to be shared with many pathogenic (+) RNA viruses.

## Teaser

CryoEM structure of CHIKV replication complex reveals multicomponent RNA synthesis nanomachine stuck in host cell plasma membrane.

## INTRODUCTION

Chikungunya virus (CHIKV, genus *Alphavirus*, family *Togaviridae*) is a mosquito-borne pathogen that has spread globally and caused a substantial health burden of acute febrile illness progressing to debilitating, often chronic polyarthritis in millions of people. In addition, CHIKV can also induce rare but lethal encephalitis (*1*). The alphavirus (+) RNA genome is ~11.8 kb in length, containing a 5′ N7-methylguanylated cap and a 3′ polyadenylated tail. It includes two coding regions: The first comprises two-thirds of the entire genome encoding four nonstructural proteins (nsPs) involved in genome replication, while the second (located downstream of the subgenomic promoter) encodes the structural proteins (C-E3-E2-6K-E1) necessary for virion assembly. Alphaviruses replicate their genomes in membrane-derived ultrastructures termed spherules that contain the negative-strand (−) RNA template, likely in the form of double-stranded RNA (dsRNA) intermediate species, and the replication complex (RC) assembled from nsPs (*2*, *3*). The RC likely creates a favorable compartment for viral RNA synthesis that minimizes the host immune response to dsRNA intermediates and possesses multifunctional enzymes capable of orchestrating rapid and efficient synthesis of genomic and subgenomic viral RNAs (*4*–*9*). nsP1 to nsP4 are structurally known to localize to the RCs and are essential to viral RNA replication through their own distinct enzymatic and nonenzymatic functions: nsP1 [Protein Data Bank (PDB) 7DOP] displays methyl- and guanylyltransferase activities required for viral RNA 5′ cap synthesis and plasma membrane (PM) anchoring ability (*10*, *11*); nsP2 consists of an N-terminal superfamily 1 RNA helicase (PDB 6JIM) and a cysteine protease at the C-terminal region (PDB 3TRK) responsible for polyprotein autoprocessing (*12*, *13*); nsP3 (PDB 3GPG) contains an adenosine diphosphate–ribosyl binding and hydrolase domain and a disordered region as the host factor interaction hub (*14*, *15*); last, nsP4 (PDB 7VB4/7F0S) is the RNA-dependent RNA polymerase (RdRp) for viral RNA synthesis (*16*, *17*). Despite having all the high-resolution individual structures of the nsP1 to nsP4, the overall architecture and assembly mechanisms of the alphavirus RC remains unresolved. The ultrastructure of the distantly related Flock House nodavirus (FHV) RC provided the first structural insight into RC assembly in the form of a dodecameric crown-shaped scaffold anchored at the neck of spherule packed with viral RNA condensate (*9*, *18*). Similarly, multimeric ring-shaped ultrastructures were recently reported in both coronavirus RC (*19*) and alphavirus nsP1 (*10*, *11*, *20*). However, these ultrastructures have yet to explain the organization of multicomponent RCs and the molecular mechanism by which RCs ultimately achieve viral RNA synthesis and transport to the cytosol.

Here, we applied complementary single-particle cryo–electron microscopy (cryo-EM) and cryo–electron tomography (cryo-ET) methods to determine the molecular architecture of the alphavirus RC. The molecular structure of the reconstituted RC core (nsP1 + 2 + 4) fits nicely into part of the active RC density obtained from subtomogram averaging of the necks of the alphaviral spherules. Together, these data provide unprecedented molecular basis of the alphavirus genome replication process, likely applicable to other (+) RNA viruses, such as coronaviruses and flaviviruses.

## RESULTS

### Reconstitution of a functional alphavirus RNA replicase

**Fig. 1.**
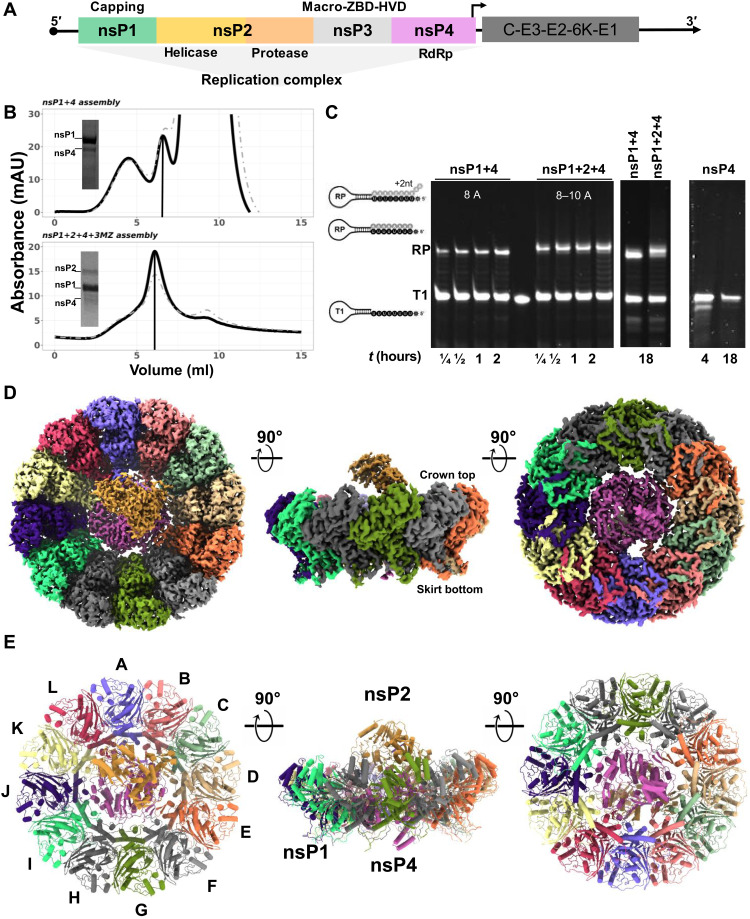
Recombinant nsPs were in vitro reconstituted via tandem purification and activated their RNA synthesis ability. (**A**) The alphavirus genome is partitioned into two open-reading frames (ORFs) where the first ORF encodes for four nsPs (nsP1 to 4) to assemble into an RC for the viral RNA replication process, while the second ORF is regulated by a 26*S* promoter (black arrow) and encodes for structural proteins (C-E3-E2-6K-E1) for virion assembly. (**B**) Top: Anion-exchange chromatography profile of nsP1 + 4 assembled from CHIKV nsP1 mutant (H37A; amino acids 1 to 535) and ONNV nsP4 (amino acids 1904 to 2514), eluted at 32 mS/cm (elution peak marked with vertical line). Bottom: Anion-exchange chromatography profile of RC core (nsP1 + 2 + 4 + 3 M) assembled from CHIKV nsP1 mutant (H37A; amino acids 1 to 535), CHIKV nsP2 (nsP2; amino acids 536 to 1333), CHIKV nsP3 macrodomain (nsP3MZ; amino acids 1334 to 1659), and ONNV nsP4 (amino acids 1904 to 2514), eluted at 29 mS/cm (elution peak marked with vertical line). mAU, milli–absorbance unit. (**C**) The RNA polymerase activity of the RCs was observed with elongation of the T1 RNA template to its RNA products (RPs; the number of ATP incorporation labeled in white letters for each RC) at several time points (0.25 to 18 hours) along with the nsP4 recombinant protein as a control. (**D** and **E**) The graphical illustrations of RC core (nsP1 + 2 + 4) architecture in map representation in (D) and molecular structure in (E) at their top view, side view, and bottom view (90° rotations, from left to right). The coloring in (D) and (E) was assigned according to their chain numbers in the RC core (nsP1 chain coloring: violet, A; salmon, B; light green, C; tan, D; orange, E; light gray, F; green, G; dark gray, H; spring green, I; indigo, J; light yellow, K; and crimson, L; nsP4 chain coloring: magenta, X; nsP2 chain coloring: peru, Y; RNA of nsP2 chain coloring: green, Z).

To reconstitute a functional alphavirus RC (Fig. 1A), we prepared soluble recombinant nsP1, nsP2, and nsP3 with C-terminal (disordered region) truncation (nsP3MZ), and nsP4 separately and ensured the enzymes were well folded and functional (refer to Materials and Methods). For this purpose, we used nsP1, nsP2, and nsP3MZ from CHIKV, and nsP4 from o’nyong-nyong virus (ONNV) because we could only obtain soluble proteins of the full-length nsP4 from ONNV but not that of CHIKV. ONNV nsP4 (referred as nsP4 hereafter) is functionally interchangeable with the closely related CHIKV nsP4 in the trans-replicase system (*21*, *22*). Using a tandem purification strategy, we successfully reconstituted two complexes: the full-length proteins of nsP1 and nsP4 (nsP1 + 4) and the full-length proteins of nsP1, nsP2, and nsP4 but not nsP3MZ (nsP1 + 2 + 4). Judging from the band intensity in the SDS–polyacrylamide gel electrophoresis (SDS-PAGE) for nsP1 + 2 + 4 purification, nsP1 is in great excess of nsP2 and nsP4 (Fig. 1B). To measure the biological functionality of the reconstituted complexes, we applied an RNA elongation assay to measure the polymerase activities of nsP4 alone, the nsP1 + 4 complex, and the nsP1 + 2 + 4 complex. Using a hairpin RNA template with 5′ octauridine overhang, the recombinant alphavirus nsP4 proteins were previously shown to have weak in vitro RNA polymerase activity (*17*). Here, the polymerase activities of the nsP4 on the same T1 RNA template were substantially enhanced upon assembly into stable complexes of nsP1 + 4 and nsP1 + 2 + 4 (Fig. 1C). In contrast to the inactive standalone nsP4, nsP1 + 4 and nsP1 + 2 + 4 complexes catalyzed the formation of the expected full-length RNA product (RP) in 15 min. The nsP1 + 2 + 4 complex generally outperformed the nsP1 + 4 complex throughout the time course. Furthermore, the nsP1 + 2 + 4 complex was able to produce not only RP but also RP + 1 nucleotide (nt) (dominant product), RP + 2 nt, and RP + polyA-tail (faint smears above the major bands in lanes labeled nsP1 + 2 + 4) as indicated in Fig. 1C. These results demonstrated that (i) nsP1 substantially promoted the polymerase activity of nsP4 upon forming the nsP1 + 4 complex, and (ii) nsP2 binding further stabilized nsP4 and thus enhanced the polymerase activity of the nsP1 + 2 + 4 complex. While nsP1 + 2 + 4 forms the core RNA replicase (referred as RC core hereafter) to catalyze the essential chemical reactions during RNA polymerization, nsP3 is likely to play additional but essential roles in viral spherule formation and RNA replication inside virus-infected cells.

### Molecular structure of the CHIKV RC core

**Fig. 2.**
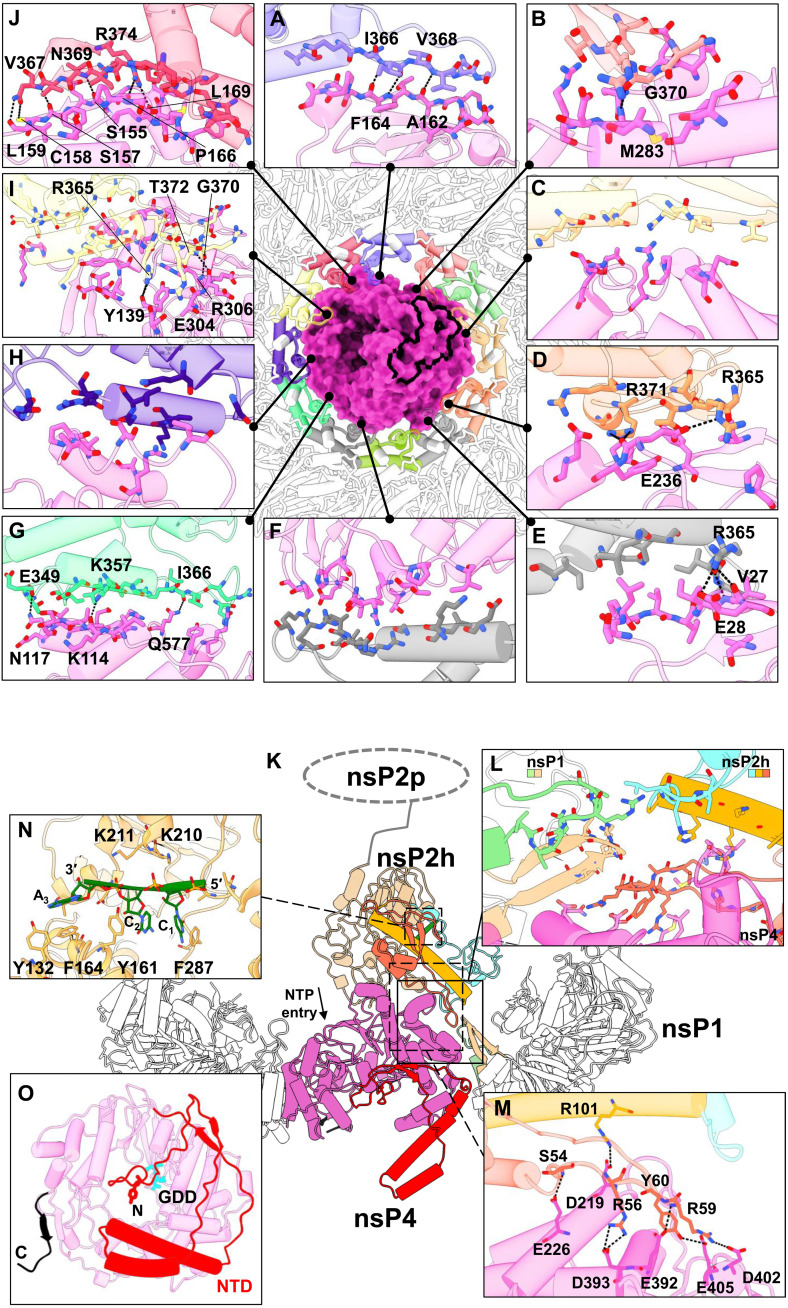
The macromolecular architecture of the RC displays a multiple interface network. (**A** to **J**) The interaction network of RC (made of nsP1 + 2 + 4 at the top view) is presented with each colored by each subunit chain for nsP1 dodecameric ring and nsP4 (surface), based on Fig. 1 chain coloring. For clarity of the interaction overview, nsP2 and its RNA ligand are hidden. Instead, the nsP2:nsP4 interface area is outlined with a thick black line. The hydrogen bonds (black dotted lines) between the individual chain of nsP1 and nsP4 are shown here in the interface blown-up view with each residue involved named and colored according to the chain. (**K**) The overall side-view impression of interfaces spanning across CHIKV RC (cartoon representations) where their three-way interactions between nsP1, nsP2, and nsP4 are shown in (**L**) a blown-up window (solid line and box). The nsP2h (amino acids 1 to 465) region is colored according to its subdomain: NTD in orange, STALK in gold, 1B in cyan, and RecA1-A2 in peru, while chains C and D of nsP1 are respectively colored in light green and tan. The unbuilt nsP2 protease (nsP2p; after amino acids 466) region is drawn here at the C terminus of nsP2h for visual guidance for (K). NTP entry site at the nsP4 motif D within the palm subdomain (magenta region) is annotated. (**M**) The hydrogen bonds (dotted lines) between the nsP2:nsP4 interface are listed on another blown-up window (dotted line and box). (**N**) The interacting residues from nsP2h and RNA (green) are labeled at a zoomed-in view (dotted line and box). (**O**) The bottom view of nsP4 showcases the spatial coordinates of its C terminus (C; black; amino acids 600 to 611) and N-terminal domain (NTD; red; amino acids 1 to 105) and the active site (named GDD; cyan stick).

To gain molecular insights into the virus replication process, we determined the cryo-EM structure of the nsP1 + 2 + 4 RC core at an overall average resolution of 2.8 Å using the 0.143 Fourier shell correlation criterion (Figs. 1 and 2, figs. S1 and S2, and table S1). The ternary complex is assembled at a stoichiometric ratio of 12:1:1, where a single copy of nsP2 is docked above a unit of nsP4 that is slotted within the central pore of the nsP1 dodecameric ring, forming a disk-shaped base with central protrusion (Fig. 1, D and E, and fig. S1C). On the basis of the nsP1 dodecamer structures solved recently (*10*, *11*, *20*), the crown top and the skirt bottom of the nsP1 dodecameric ring face the cytoplasm and the spherule, respectively. In the RC core, nsP2 extends toward the cytoplasmic side from the nsP1 + 4 disk (Fig. 1, D and E). The total buried area between the nsP1 ring and nsP4 is 4804 Å^2^, indicating that the nsP1:nsP4 interface is highly stable (fig. S3A). Intriguingly, the nsP1 ring acts as a chaperone to interact with and stabilize the otherwise highly dynamic nsP4 (*16*, *17*). Specifically, the nsP1 ring uses the oligomeric α-helical bundles (amino acids 335 to 364) on the inner face to hold nsP4 at the center of the dodecameric ring and recruits the flexible loops (amino acids 365 to 380, disordered in the structures of nsP1 alone and hereafter named as the hooking loop) from 10 of the 12 members of the nsP1 ring to tightly hook onto nsP4 (Figs. 1 and 2 and figs. S1, D and E, S2, and S3). The 10 unique nsP1:nsP4 interfaces are mapped to the surfaces of the N-terminal domain (NTD) and fingers, palm, and thumb domains of nsP4, which are highly conserved across the alphaviruses (fig. S3, D and E). The hydrogen bonding occurs in 7 of the 10 nsP1:nsP4 interfaces (Fig. 2, A to J). We identified a putative molecular channel that is formed between nsP4 and nsP1 (chains A and B) to connect the spherule compartment to the cytoplasm (fig. S2E). In particular, nsP4 is oriented such that its RNA entry and exit pockets, which are conserved among all (+) RNA virus RdRps, and its NTD are all facing the spherule side of nsP1, whereas its nucleoside triphosphate (NTP) entry tunnel has full exposure to the cytoplasm (Fig. 2, K and O). In consequence, the C12 symmetry of nsP1 is disrupted upon binding to nsP4. The C-terminal tail (amino acids 477 to 535) of nsP1 remains disordered, suggesting that these residues are not involved in any interaction with nsP2 or nsP4 (Fig. 1, D and E, and fig. S1E).

The interface between nsP2 and the nsP1 + 4 subcomplex is mainly between the N-terminal NTD-Stalk region of nsP2 (helicase domain) and the palm subdomain of nsP4 with a buried area of 906 Å^2^ (Figs. 1 and 2 and figs. S2A and S3). In addition, nsP2 is close to the tips of the hooking loops of nsP1 (chains C and D), forming a three-way interaction network between nsP1, nsP2, and nsP4 (Fig. 2L). A flexible loop on nsP2 NTD (amino acids 56 to 65) that was disordered in the crystal structure of CHIKV nsP2 helicase (PDB 6JIM) (*12*) folds into a hairpin structure, which slips into the interspace between nsP4 and the hooking loop of nsP1 (chains C and D) (Fig. 2, L and M, and fig. S3). Conserved residues R56, R59, Y60, and R101 from this nsP2 hairpin structure established several hydrogen bonds with conserved residues D219, E392, D393, D402, and E405 from nsP4 with proximal contacts to V368, N369, and G370 from nsP1 (chain D) (Fig. 2, L and M, and fig. S3). While the interface between nsP2 and nsP1 + 4 is well resolved in the cryo-EM map, the rest of the nsP2 density, including RecA-like domains 1 and 2 of the helicase core (nsP2h) and the C-terminal protease region, becomes more flexible and gradually unresolved (Fig. 2K and figs. S1 and S3). Nonetheless, we identified and built a short single-stranded RNA (ssRNA) of 3 nt within the nsP2h RNA binding groove, in a similar manner to its reported crystal structure (Fig. 2N and fig. S2B) (*12*). The nsP2h-bound RNA may represent the product RNA being exported from the viral spherule before being transported either to the ribosome for translation or to the capsid proteins for virion assembly.

The nsP4 RdRp resides at the center of the RC core, well stabilized by the surrounding nsP1 and nsP2. Almost all 611 residues of the nsP4 are well resolved in the cryo-EM density map, which includes the intrinsically disordered NTD (amino acids 1 to 105) and those disordered regions from the fingers subdomain (subdivided into the index, middle, ring, and pinky fingers) not captured in the crystal structures of the alphavirus nsP4 RdRps from Ross River virus (PDB 7F0S) and Sindbis virus (PDB 7VB4) (Figs. 1, D and E, and 2O and figs. S2 and S4) (*17*). The NTD of nsP4 is composed of several characteristic structural elements (Fig. 2O and figs. S2D and S4A): (i) The N-terminal tip (amino acids 1 to 25) starts from the alphavirus-conserved Y1 residue and is inserted into the central RNA binding groove; (ii) one helix-turn-helix motif (amino acids 42 to 84) interacts with the pinky finger motif and extends toward the spherule space; and (iii) anti-parallel β strands (amino acids 26 to 30 and 98 to 102) along with the surrounding unstructured loops wrap around the RdRp core domain. The C-terminal tail of nsP4 is also well folded and points to the spherule side of the RC core. Overall, the well-folded nsP4 structure within the RC core explains the enhanced polymerase activities (Fig. 1C) and reveals both unique and conserved molecular features when compared to the structurally related RdRps from other (+) RNA viruses (fig. S4).

### Molecular organization of CHIKV RC in situ

**Fig. 3.**
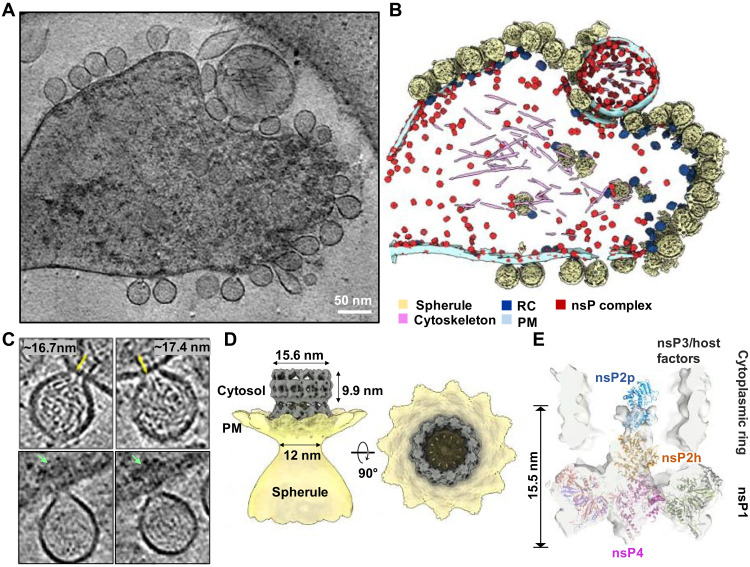
CHIKV RNA replication spherule structures revealed by cryo-ET. (**A**) Tomographic slice of cell periphery depicting CHIKV RNA replication spherules at the PM. Scale bar, 50 nm. (**B**) Corresponding 3D segmentation of cellular features. See also movie S1. (**C**) Snapshot of the individual spherules. Yellow arrows measure the ordered density within the center of the cytoplasmic ring. Green arrows mark the additional associated density proximal to the RC cytoplasmic ring. (**D**) CHIKV spherule 3D volume is determined by subtomogram averaging with imposed C12 symmetry. (**E**) The RC core complex (nsP1 + 2 + 4) is fitted into the C1 subtomogram average map of the RC. A cytoplasmic ring as observed in (C), likely made of nsP3, RNA, and host factors remain loosely connected to the nsP1 ring, which is bound at the neck of the spherule. The extra density above the nsP2h region is likely to be the C-terminal protease of nsP2, stabilized or restrained by the cytoplasmic ring.

To validate the biological relevance of the in vitro reconstituted alphavirus RC core and understand its functions in the native RC in virus-infected cells, we performed cryo-ET of CHIKV-infected cells at 8 hours post-infection (h.p.i.). At this time point, all RCs should be matured with fully processed nsPs and dsRNA replication intermediate (*23*, *24*). Unlike spherules of other alphaviruses that form at the PM and are subsequently internalized to cytopathic vacuoles via phosphatidylinositol 3-kinase–Akt activation, CHIKV spherules remain at the PM without internalization (*25*). Taking advantage of the predominant location of CHIKV spherules at the PM, we collected tomographic tilt series of the periphery of the CHIKV-infected cells, which were decorated with many spherules filled with ball-of-yarn–like densities (Fig. 3, A and B). The swirling densities inside the spherules likely correspond to dsRNA intermediates. CHIKV spherules bulge outward from the PM with membrane-associated macromolecular complexes, assumed to be active RCs, gating the spherule neck on the cytoplasmic side of the PM (Fig. 3, A and B). Spherules are generally balloon-shaped with an average short and long axis of 63.1 ± 4.7 and 79.0 ± 6.5 nm (*N* = 101 spherules), respectively. Proximal to the spherules, dense condensates in the cytosol are often observed proximal to RCs at the neck (Fig. 3C). Such density can be attributed to RNA, RNA/capsid mixture, and/or cell host factors recruited to active replication sites (*4*, *26*, *27*).

Subtomogram averaging of the spherule neck regions revealed a 12-fold symmetric complex at 7.3 Å resolution, consisting of a PM-associated crown ring connected to a larger, smooth ring on the cytoplasmic side (Fig. 3D and fig. S5, A to C). The PM-associated crown ring closely matches the reported cryo-EM structures of the recombinant nsP1 dodecamer (*10*, *11*, *20*). While the crown ring contains a plug-like density in the 12-fold symmetric average, the cytoplasmic ring features a cylinder-like density with a notably wide and mostly empty cavity (15.6-nm diameter and ~10-nm height) (Fig. 3D). Together, the RC extends approximately 17 nm from the bottom of the crown ring to the top of the cytoplasmic ring (Fig. 3C). In tomogram slices, contiguous linear density was observed, which we attributed to RNA transported from the spherule through the crown and the center of the cytoplasmic ring. Such density was blurred out in the average by the imposed 12-fold symmetry. The two rings in the subtomogram average are linked with a fixed orientation by thin density, potentially nsP1 C-terminal helices that are unresolved in the nsP1 dodecamer cryo-EM structures (figs. S1E, S5, and S6) (*10*, *11*, *20*). Future work is warranted to determine how the two rings assemble into the active RC and the other cofactors that potentially guide their assembly.

We then determined an asymmetric reconstruction of the RC, revealing that the plug density in the center of the crown is positioned asymmetrically with a small gap on one side (Fig. 3E and fig. S6). This is consistent with the structure of the active nsP4 RdRp in the RC core complex that contacts 10 of 12 nsP1 hooking loops (Figs. 1 and 2). Above the crown, notable density was resolved in the center of the cytoplasmic ring. The abovementioned RC core complex (Fig. 1, D and E) docked nicely into this cryo-ET reconstruction, while additional density above the nsP2h domain is likely the nsP2 protease domain (nsP2p) that is unresolved in the reconstituted RC core complex (Fig. 3E). It is possible that the cytoplasmic ring chamber confines this highly flexible region of nsP2 (*13*) and/or nsP2p forms loose interactions with RNA and/or the interior of the ring that stabilizes its conformation in the mature RC. Since nsP3 is missing from the reconstituted RC core complex and known to localize to the active RC (*28*), we postulate that the cytoplasmic ring in our reconstruction comprises nsP3, newly synthesized viral RNA, and possible host factors. In total, these reconstructions reveal the composition of the viral RC core in the native cellular context and suggest the overall molecular architecture of this elegant nanomachine for viral RNA production.

### Excess nsPs form membrane-associated nonreplicative complexes

**Fig. 4.**
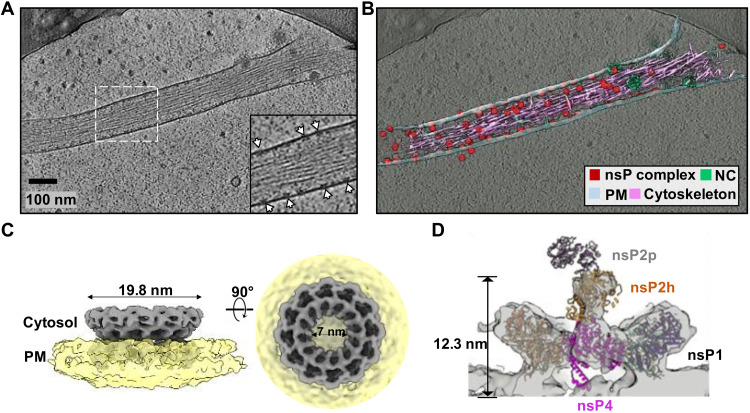
Inactive nsP complex induced membrane protrusion revealed by cryoET. (**A**) Tomographic slice of cell periphery depicting a filopodia-like membrane protrusion structure extended from the PM. White arrows point to the membrane-associated nsP complexes. Scale bar, 100 nm. (**B**) Corresponding 3D segmentation of cellular features. See also movie S2. (**C**) CHIKV nsP complex 3D volume determined by subtomogram averaging with imposed C12 symmetry. (**D**) The RC core complex (nsP1 + 2 + 4) is fitted into the C1 subtomogram average map of the nsP complex. Note that the cytoplasmic ring as observed in Fig. 3C is absent in this inactive nsP complex. Consequently, there is no density observed for the C-terminal protease of nsP2.

In addition to the RCs positioned at the neck of spherules, we observed many ring-like complexes smaller than RCs docked to the inner leaflet of the PM in the absence of RNA or membrane invaginations. These macromolecular complexes were especially numerous on thin cell extensions with underlying bundled cytoskeleton filaments (Fig. 4, A and B, particles in red named the nsP complex, fig. S7, A and B), reminiscent of the reported distribution of nsP1 in filopodia (*29*, *30*). Occasionally, we also observed extracellular vesicles of various sizes that were decorated with such complexes on the inner leaflet (Fig. 3, A and B, and fig. S7B). To reveal the molecular identity of these complexes, we determined a subtomogram average of more than 5000 extracted particles to ~10 Å resolution (fig. S5, D to F). The reconstruction revealed a 12-fold crown ring ~20 nm in diameter with a 7-nm central pore that closely matches the nsP1 dodecamer reported (Fig. 4C) (*10*, *11*, *20*). We then considered whether these rings were nsP1 alone or comprised multiple assembly states of nsPs. To answer this, we determined an asymmetric reconstruction of the rings, revealing asymmetric density plugging the center of the crown and density positioned above the center of the ring scaffold on the cytoplasmic side. This density corresponded nicely to the RC core complex described above (Fig. 4D and Fig. 1, D and E). Noticeably, the nsP2 attributed density only included the helicase domain, while the protease region and the large cytoplasmic ring of the RC were not detected. To determine whether there were any nsP1 rings alone, we performed three-dimensional (3D) classification on this asymmetric complex, which revealed that all the rings were composed of RC core members: nsP1 + 2 + 4 (fig. S7C). Therefore, while expression of nsP1 alone is apparently responsible for the induction of thin extensions (*29*, *30*), it is likely achieved via the RC core complex formations in the virus-infected cells, although we cannot exclude that nsP1 functions in a form not detected in our study. Since our time point of imaging (8 h.p.i.) is considered late in the CHIKV replication cycle, the production of large amounts of cleaved nsPs likely results in extreme molar excess of the nsP1 + 2 + 4 complex relative to template strand viral RNAs. Therefore, we consider such complexes nonreplicative because of the absence of the cytoplasmic ring and the spherule (and the template RNA).

## DISCUSSION

In this study, we performed a multiscale study for the structural and functional characterization of the CHIKV replication machinery in vitro and in the infected cell using combinatory cryo-EM methods. Our work provides an integrated molecular view of the structure of a central core portion of the alphavirus nsP assembly within the RCs. The nsP1 dodecameric ring of nsP1 + 2 + 4 RC core architecture did not deviate much from the previously published nsP1 ring structure (PDB 7DOP) (*10*) within a root mean square deviation of 0.89 Å (416 Cα pairs of each nsP1 subunit) except for the additional presence of conformationally stabilized hooking loops as a result of interactions with nsP4 (fig. S1, D and E). Furthermore, the RC core may recruit nsP3, viral RNA, and host cofactors to construct the mature viral RNA production factory—a spherule on the PM.

Viral RdRps play a central role in the virus replication process and serve as a top target for antiviral development (*31*, *32*). Unlike other viral RdRps from the *Piconaviridae* (*33*) and *Flaviviridae* (*34*) that possess relatively good in vitro RNA polymerase activity, we showed that alphavirus nsP4 gets activated upon proper folding within the central pore of nsP1 dodecameric ring. nsP2 binding further boosts the nsP4 enzymatic activities and seems to increase the terminal adenylyltransferase activity, which is essential for the 3′-end polyadenylations of the genomic and subgenomic RNAs (Fig. 1). This is functionally similar to the coronavirus nsp12 RdRp, which requires cofactors nsp7 and nsp8 for proper binding to the RNA substrate to gain decent polymerase activity (*35*–*37*). On the basis of this nsP4 localization, it is intriguing to speculate that density in the center of the coronavirus nsp3 hexamer, unresolved in the subtomogram averages with applied rotational symmetry, represents the coronavirus RdRp (fig. S6) (*19*). This conserved RC architecture would provide an elegant solution to the mechanisms of RNA synthesis, immediate capping of nascent RNAs, and sealing the membrane microcompartment while exporting RNAs to the cytosol for translation and packaging into virions.

**Fig. 5.**
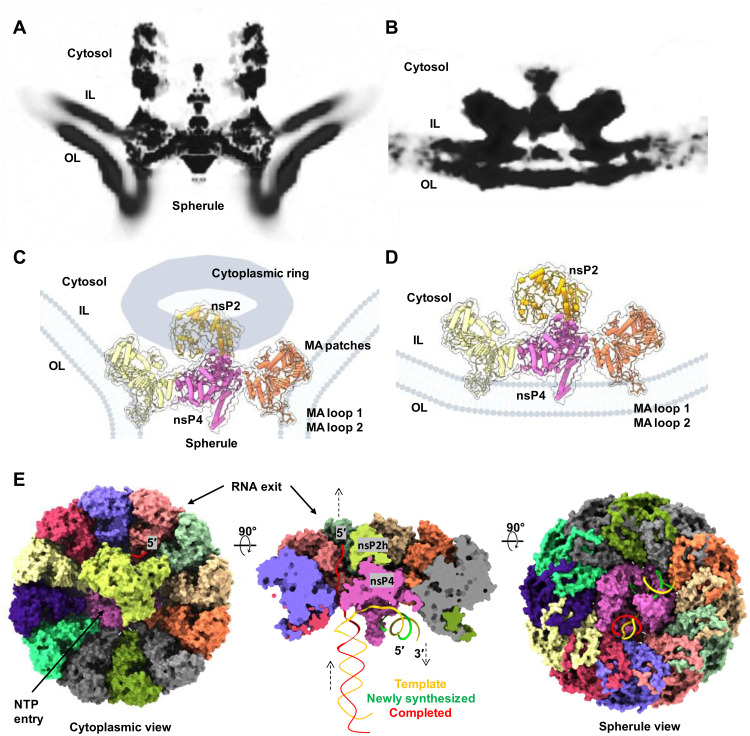
Remodeling of the host membrane is coupled to assembly of active viral RC. (**A**) A central slice view of the tomography volume at the active viral RC spherule highlights the curved PM at the nsP1 contact sites [membrane association loops 1 and 2 (MA loops 1 and 2) from the bottom of the nsP1 ring and newly identified MA patches from the nsP1 upper ring] as shown in (**C**). In contrast, (**B**) shows the central slice view of the tomography volume at the inactive viral nsP complex, which is simply docked to the inner leaflet of the PM, using the MA loops 1 and 2 on nsP1 ring in (**D**). (**E**) Molecular model of how the viral RC core complex facilitates viral (+) RNA replication. The dsRNA replication fork is modeled and colored. Template RNA, i.e., antigenomic (−) RNA is in yellow, newly synthesized progeny RNA (or subgenomic RNA transcript) is in green, and the RNA in red is the completed product RNA to be exported to the cytoplasm for translation and virion assembly. NTP entry tunnel on nsP4 polymerase and the putative RNA exit tunnel are labeled (also refer to fig. S2, E and F).

The NTD subdomain of nsP4 is unique with its N terminus inserted into the RNA binding groove, which is equivalent to the priming loop of dengue virus nonstructural protein 5 RdRp (Fig. 2O and fig. S4) (*34*, *38*, *39*). As a result, the N terminus of nsP4 NTD needs to move away to allow the binding of the dsRNA elongation substrate (*40*). The helix-turn-helix motif in nsP4 NTD that protruded into the spherule lumen may also provide an additional binding footprint for supporting dsRNA binding. The putative molecular channel between nsP4 and nsP1 (chains A and B) interface may serve as a bidirectional ssRNA translocation channel to export RPs to the cytosol as well as to import the parental viral (+) RNA for (−) RNA synthesis (Fig. 5 and fig. S2, E and F). The alphavirus-unique nsP4 NTD could also have adaptively evolved to organize the flexible loops of NTD and fingers subdomains into an extensive surface for enabling nsP1:nsP4 interactions. Only one copy of nsP2 is found to bind to nsP4 through its NTD region at the cytoplasmic side (Figs. 1, D and E, and 2K). This molecule of nsP2 improves the polymerase activity on the short RNA substrate, likely via binding and stabilizing the active conformation of nsP4 (Figs. 1 and 2). As a superfamily 1 RNA helicase, nsP2h translocates along ssRNA in a 5′→3′ direction (*41*). Given its relative location and orientation to the nsP4 polymerase, nsP2h may function by pulling out the product RNA from the spherule analogous to a pulley system, a hypothesis that requires further study.

While nsP1 expression alone was reported to drive cell morphological change and induce the formation of thin extensions, the molecular organization of nsP1 and its mechanism of action in virus-infected cells is unclear (*42*–*44*). Here, we demonstrated that at the molecular level, nsP1 rings in the nonreplicative form of nsP1 + 2 + 4 RC core complex are present in extremely high concentrations in thin extensions, with each inducing a small and local positive curvature on the membrane (Figs. 4, A to C, and 5, B and D). It is conceivable that in total, these nsP1-induced membrane deformations stabilize high membrane curvature to drive formations of thin extensions (*30*, *43*). How nsP1 induces actin remodeling remains to be determined. One interesting observation in this study is that these nsP1 + 2 + 4 RC core complexes seal the active RNA replication spherules only when they assemble with cytoplasmic rings into active holo-RCs. Unlike RC core alone docking at the PM, the RC core in the holo-RC supports a high negative curvature at the spherule neck via extensive binding of nsP1 to the membrane (Fig. 5, A and C). We identified additional membrane association (MA) patches on the peripheral side of the nsP1 ring (Figs. 3E and 5, A and C, and fig. S8). Together, these MA loops and the MA patches around the nsP1 ring maintain a tight engagement with the negatively bent PM (Fig. 5, A and C, and fig. S8), which seems to be functionally equivalent to the transmembrane domains of the nsp3 from coronavirus and the N-terminal mitochondrial membrane localization sequence of FHV protein A (fig. S6) (*19*, *45*). It is not known whether the stable yet inactive nsP1 + 2 + 4 RC core complexes may be activated by recruiting the nsP3-cytoplasmic ring with viral RNA and potentially replace those functional RCs prone to degradation by host response. On the basis of nsP1:nsP2 stoichiometry in the RC, extra nsP2 protein exists that is not incorporated into the viral RC. It is reported that nsP2 from the Old World alphaviruses may be transported into the nucleus to shut off host cell transcription (*46*). nsP2 from the New World alphaviruses may remain predominantly inside the cytoplasm and help modulate virus-host interaction by interacting with host factors (*47*). For example, Venezuela equine encephalitis virus nsP2 was demonstrated to induce host translational shutoff, although the mechanism of this activity is not well defined yet (*48*). Future work is warranted to address these questions.

On the basis of the current knowledge, we generated a model for CHIKV (+) RNA replication (Fig. 5E). The spherules are thought to contain viral dsRNA intermediates, comprising the (−) RNA template and (+) nascent RNA, and export replicated/transcribed ssRNA species to the cytosol for translation or virion packaging. During viral RNA replication, the synthesis of a nascent RP and the export of the completed product RNA from the last replication are coupled, thus reaching an equilibrium state with a consistent amount of RNA that maintains the 3D volume of a mature spherule. Capping the 5′-end of the product RNAs can be conveniently catalyzed by nsP2 and nsP1 immediately after they are exported to the cytosolic surface of the RC. The cytoplasmic ring of the RC that is attached above its RC core complex seems to be essential in maintaining the continuous material exchange between the spherule and the host cell cytoplasm (Figs. 3, C to E, and 5, A and C). Overall, the CHIKV RC structure provides the molecular basis of viral RNA replication and serves as a useful tool for antiviral development against alphaviruses and other (+) RNA viruses.

## MATERIALS AND METHODS

### Recombinant protein productions of alphavirus nsPs

The preparation of nsP1 was adapted from (*10*, *11*). The gene cassette of CHIKV nsP1 (amino acids 1 to 535) comprises affinity fusion tags of StrepII-3XFLAG after A516 residue and mutation of H37A, created using In-Fusion Snap Assembly kit (Takara Bio) and subcloned into enhanced green fluorescent protein plasmid (pEGFP)-C1 (Clontech) with eGFP eliminated (*10*). This nsP1 plasmid was expressed transiently in the Expi293 Expression System (Thermo Fisher Scientific) by PEI MAX (Polysciences) transfection and harvested at 4000*g* after 5 days. The protein expression was boosted by 10 mM sodium butyrate 16 to 20 hours after transfection. The cell pellet was subsequently lysed in lysis buffer WI [50 mM Hepes (pH 8.0), 150 mM NaCl, and 0.5 mM tris(2-carboxyethyl)phosphine (TCEP)] with 1 mM phenylmethylsulfonyl fluoride (PMSF) protease inhibitor (Sigma-Aldrich), 1% *n*-dodecyl-β-d-maltoside (Exceedbio), and BioLock solution (70 mU/ml; IBA Lifesciences) on a rotator at 4°C for 1.5 hours and pulse-sonicated for 5 min. The lysate was clarified with 100,000*g* centrifugation at 4°C in XPN-100 ultracentrifuge (Beckman Coulter). The Strep-Tactin Sepharose slurry beads (IBA Lifesciences; named strep-bead hereafter) were incubated in Econo-Pac column (Bio-Rad) with the clarified supernatant. The nsP1-bound strep-bead was equilibrated to assembly buffer WII [50 mM Hepes, 150 mM NaCl, 2.5 mM MgCl_2_, 0.5 mM TCEP, 0.008% Glyco-diosgenin (GDN), 5% glycerol, and 2.5% sucrose (~pH 8)] in 5 column volume (CV) wash for downstream RC assemblies and RNA polymerase assay (refer to below).

The full-length nsP2 (nsP2; amino acids 536 to 1333) and nsP2 helicase domain (nsP2h; amino acids 536 to 1000) were both subcloned into pSUMO-LIC expression vector carrying N-terminal hexa-histidine tag fused with cleavable SUMO (N-His_6_-SUMO) tag and recombinantly purified as described in (*13*) and (*12*), respectively.

The nsP3MZ gene (amino acids 1334 to 1659 of nsP3, which includes the macro domain and the zinc ion binding domain) was cloned from the same CHIKV cDNA described in (*10*) into pSUMO-LIC expression vector with N-His_6_-SUMO tag. The C-terminal disordered region of nsP3 was excluded for this cloning for the nsP3MZ solubility. The nsP3MZ was overexpressed in Rosetta-2 T1R *Escherichia coli* strain and cultured in Luria-broth Miller media (Axil Scientific) supplemented with 2.5% glycerol and then induced at 0.5 mM isopropyl-β-d-thiogalactoside (Gold Biotechnology) after a measured OD_600_ (optical density at 600 nm) of ~0.8 for 18 hours at 18°C. The following purification process for nsP3MZ was maintained throughout at 4°C. The purification started with harvesting the bacterial pellet at 4000 rpm followed by lysis with buffer A [2× phosphate-buffered saline (PBS), adjusted to 500 mM NaCl, 10% glycerol, and 5 mM β-mercaptoethanol] supplemented with 1 mM PMSF in a PANDAPLUS2000 (GEA) homogenizer. The supernatant obtained from 100,000*g* ultracentrifugation was incubated with Ni-NTA slurry beads (Bio Basic) for 1 hour. The nsP3MZ-bound Ni-nitrilotriacetic acid (NTA) was washed for 20 CV of buffer A and eluted in buffer B (buffer A with 300 mM imidazole). The eluant was cleaved with SUMO protease (at 10:1 w/w ratio) for 16 hours in 0.5× buffer B. Then, the tag-removed nsP3MZ was purified subsequently in HiLoad 16/600 Superdex 200 pg column (Cytiva) in buffer C [25 mM Hepes (pH7.5), 150 mM NaCl, 5% glycerol, and 2 mM dithiothreitol] as a monomer. The monomeric peak fractions were concentrated to desired concentration in buffer C to flash freeze for long-term storage at −80°C till required.

The full-length nsP4 gene (amino acids 1904 to 2514) was sourced from ONNV (AF079456.1; a gift from A. Merits) and cloned into pSUMO-LIC expression vector with N-His_6_-SUMO for recombinant protein preparation using the same bacterial expression and purification method as described in (*17*). ONNV nsP4 has a sequence similarity of ~91% to CHIKV.

### RNA polymerase assay

The RNA polymerase assay is optimized and uses the same T1 RNA template from (*17*). All assay reactions were performed with 1 μM protein (nsP1 + 4, nsP1 + 2 + 4 and nsP4) and 50 nM T1 RNA template in 0.5× assay buffer [1× buffer Z: 25 mM Hepes, 75 mM NaCl, 20 mM KCl, 5 mM Mg(CH_3_COO)_2_, 2.5 mM MnCl_2_, 5 mM TCEP, 0.01% GDN, NEB murine RNase inhibitor (0.5 U/μl), and 1.25% sucrose (~pH 8)] prepared in nuclease-free water (HyClone). The reactions were initiated with 2 mM adenosine triphosphate (ATP) and quenched in a discontinuous time point manner for 0.2 to 18 hours after incubating at ~25°C in dark. The reactions were quenched with equal volume of denaturing RNA loading dye (95% formamide, 0.02% SDS, 0.01% xylene cyanol, 0.02% bromophenol blue, and 1 mM EDTA) to run on 17.5% denaturing urea-PAGE at 220 V for 1.5 to 2 hours. The urea-PAGE was imaged via Bio-Rad Gel-Doc XR+ Imager using Fluorescence Blot ALEX488 for analysis.

### In vitro transcription RNA synthesis

T2 RNA was designed to have a central double-stranded region of 50 base pairs and flanking 5′ overhangs. To synthesize T2 RNA with in vitro transcription (IVT), a DNA template with T7 promoter (5′TAATACGACTCACTATA 3′) was ordered from Integrated DNA Technologies to perform IVT according to manufacturer recommendation from Hiscribe T7 High Yield RNA Synthesis Kit (NEB). The IVT product was purified via the NEB-recommended phenol-chloroform extraction method and Monarch RNA Cleanup Kits (NEB).

T2 RNA sequences:

Sense strand: 5′GGGAUAAUGCAAGCAUACCGAUCUUCCAACGUUUCUCCGAACCCACAGGGACGUAGGAGAUGUUAUUUUGUUUUUAAUAUUUC3′

Antisense strand: 5′GGGAUAAUGCAAGCAUACCGAUCUUCCAACGUUGAAAUAUUAAAAACAAAAUAACAUCUCCUACGUCCCUGUGGGUUCGGAGA3′

### In vitro reconstitution for RC assemblies

All in vitro reconstitutions and sample preparations were done at 4°C unless otherwise mentioned. The CHIKV-nsP1–immobilized strep-bead was incubated with the desired combinations of nsPs (CHIKV nsP2 and nsP3, and ONNV nsP4) to produce RC assemblies: nsP1 + 2 + 4 and nsP1 + 4. The strep-beads consisting of RC assemblies were eluted in the abovementioned buffer WII supplemented with 10 mM desthiobiotin at ~pH 8 and loaded directly to a HiTrap Q-FF column (Cytiva) for linear elution from 0.15 M NaCl to 1 M NaCl. The eluants were concentrated at 3000 to 7000*g* in Amicon Ultra (Merck) and/or Pierce (Thermo Fisher Scientific) protein concentrators of 100 kDa molecular weight cutoff (MWCO) for the abovementioned RNA polymerase assay and structural studies. The RCs assembled at affinity pulldown and anion-exchange chromatography were fractionated and sampled for negative-staining transmission electron microscopy (TEM) on carbon-coated copper 300-mesh grids (catalog no. CF300-Cu, EMS) for screening and optimization of RC reconstitutions.

### Single-particle cryo-EM sample preparation

The nsP1 + 2 + 4 RC assembly was mixed with T2 RNA and NTP in 1× buffer Z at 1:10:100 molar ratio for 1 hour at ~25°C before concentrating down to ~30 μl. The cryo-EM grid preparation (including single-layer graphene grid fabrication) was adapted from (*10*, *11*, *49*). Before sample application, the graphene-covered R1.2/1.3 copper/gold 300-mesh grids (Quantifoil) were glow-discharged for 10 s at low radio frequency power setting in high-vacuum plasma cleaner. About 5 to 6 μl of the sample (estimated at ~0.2 to 0.5 mg/ml concentration) was applied to grids. The samples were preincubated on the grid for ~2 min within 100% humidified and 4°C chilled Vitrobot Mark IV and plunge-frozen in liquified ethane after blotting for 3 to 5 s at a blot force of −2. The grids were clipped and stored under a liquid nitrogen setting till screening and data collection.

### Single-particle cryo-EM data collection, processing, and analysis

The grids were screened at Thermo Fisher Scientific 200 kV Arctica. The data collections were conducted at Thermo Fisher Scientific 300 kV Titan Krios equipped with a Gatan K2 detector in EPU counting mode at a pixel size of 1.1 Å. Data collection parameters are detailed in table S1.

The overview of cryo-EM data processing workflow is illustrated in fig. S1. The movies recorded for all cryo-EM datasets were preprocessed in Warp (*50*) for motion correction, in a patch of 5 × 5. Then, the motion-corrected micrographs were imported into cryoSPARC (v3.3.1) (*51*) and further preprocessed with its Patch CTF module. The preprocessed micrographs were manually analyzed and selected for those with CTF estimated resolution less than 4 Å. A set of 2D references was generated using nsP1 map [The Electron Microscopy Data Bank (EMDB) ID: EMD-30796] (*10*) for the template picker for particle picking. The extracted particles were subjected to two to three rounds of 2D classifications to select for the best set of 2D class averages, estimated at 7 Å or less, for ab initio reconstructions first, if necessary, and followed by subsequent combinations of heterogenous refinements (2 to 3 classes) and 3D classifications (5 to 10 classes). The best 3D volume was selected for nonuniform refinement and uniform refinement at no symmetry applied (C1 symmetry) with ctf and defocus optimizations and optionally with Ewald sphere corrections (*52*). These maps were refined to the range of 2.4 to 2.8 Å and reasonable Guinier plot *B*-factor value for evaluations in both cryoSPARC and UCSF ChimeraX 1.3 (*51*, *53*, *54*). The PDB structures of nsP1, nsP2h, and nsP4 (PDB 7DOP, 6JIM, 7VB4, and 7F0S) (*10*, *12*, *17*) were guided/fitted into the refined maps with models generated from DeepTracer (*55*) and AlphaFold2 (*56*, *57*). The fitted structures were next exported to Coot (WinCoot 0.9.7 or Coot 0.9.5) (*58*) for chain morphing/refinement and manual building of protein chains and ligands. The built structures were refined in real-space refinement mode in Phenix (version 1.19.2) (*59*) to generate table S1 to summarize all refinement statistics. In addition, table S1 includes *Q*-score calculation (*60*) to measure the resolvability of the cryo-EM map at various regions. The graphical illustrations and molecular analyses were prepared in ChimeraX (*53*, *54*). All depictions of hydrogen bonds in less than 3 Å bond length were considered, while the other molecular contacts were considered within ~4-Å distance. Multiple sequence alignment was conducted on the MUSCLE (*61*) server and present as Weblogo3 (*62*, *63*) sequence conservation.

### Cell culture and virus strain

The human bone epithelial cell line U2OS (catalog no. HTB-96) is a female cell line purchased from the American Type Culture Collection (ATCC). Hamster fibroblast cell line BHK21 cells (catalog no. CCL-10) were purchased from ATCC. Cells were maintained at 37°C with 5% humidified CO_2_ in Dulbecco’s modified Eagle’s medium (Invitrogen) supplemented with penicillin and streptomycin, 10 mM Hepes, nonessential amino acids, and 10% fetal bovine serum (HyClone). CHIKV vaccine strain 181/clone 25 (CHIKV-181) was amplified in BHK21 cells. CHIKV vaccine strain 181/clone 25 (CHIKV-181) (catalog no. NR-13222) was obtained from BEI Resources, National Institute of Allergy and Infectious Diseases, National Institutes of Health, and amplified in BHK21 cells.

### Cellular cryo-ET sample preparation

U2OS cells grown on fibronectin-coated gold 200 mesh R2/2 grids (Quantifoil) were infected with CHIKV-181 at a multiplicity of infection of 50 for an incubation period of 8 hours. The grids were then washed with PBS and a solution of 10 nm of bovine serum albumin gold tracer (catalog no. 25486, EMS) was added directly before vitrification. Grids were blotted and plunged into liquid ethane using the LEICA EMGP plunge freezer device. Grids were stored under liquid nitrogen conditions until TEM data collection.

### Cryo-ET data collection and 3D reconstruction

Grids of vitrified virus-infected cells were imaged on a Talos Arctica (Thermo Fisher Scientific) operated at 200 kV with a post-column energy filter (20 eV) and a K2 Summit detector with a calibrated pixel size of 3.54 Å. Single-axis, bidirectional tilt series were collected using SerialEM software (*64*) with low-dose settings and a defocus range of −3 to −5.5 μm. The total average dose at the specimen was 90e^−^/A^2^, distributed over 51 tilt images, covering an angular range of −50° to +50° with an angular increment of 2°. Motion between frames of each tilt image in the tilt series was corrected using patch-tracking in MotionCor2 software (*65*). Tilt images were compiled, automatically aligned, and reconstructed into 3D tomograms using EMAN2 software (*66*, *67*). In total, 146 tomograms were judged as sufficient quality for further subvolume analysis.

### Subvolume averaging and analysis

To generate an initial model of the RC at the base of RNA spherules, 50 high–signal-to-noise ratio (SNR) particles in different orientations were picked from tomograms at ×4 binning. These RC particles were input to the EMAN2 initial model generation program, performed in two steps. First, particles were iteratively aligned with C1 symmetry for three iterations and aligned to the symmetry axis. Then, an additional five iterations were performed, applying C12 symmetry to produce an initial model with low-resolution features of the spherule base, PM, and RC. This initial model was filtered to 40 Å resolution and used for subsequent 3D subtomogram refinement of the full dataset. Subtomogram averaging was then performed with 724 RC particles at ×1 binning (3.54 Å per pixel) from 49 tomograms while applying C12 symmetry with a loose mask around RC protein density to exclude the surrounding membrane from alignment. Afterward, subtomogram refinement and additional sub-tilt refinement of particle translation and rotation produced a final RC map at 7.3 Å resolution (Fig. 3D and fig. S6). To determine a C1 reconstruction of the RC, the 12 possible asymmetric orientations based on the previous refinement with C12 symmetry were iteratively searched using the *breaksym* option in EMAN2 subtomogram refinement. After 10 iterations, this resulted in a converged map with asymmetric density features in the central region of the RC shown in Fig. 3E.

In a similar way to RCs, 100 high-SNR particles appearing as small rings on the inner leaflet of the PM were manually picked from tomograms and subjected to EMAN2 initial model generation at ×4 binning. After three iterations of C1 alignment, with the result aligned to the symmetry axis, and five additional iterations applying C12 symmetry, a low-resolution map displayed a ring-like structure docked to a membrane. This map was again blurred to 40-Å resolution and used as the input for five iterations of subtomogram refinement of 5704 ring particles at ×1 binning (3.54 Å per pixel) with C12 symmetry applied. A soft mask was applied to focus alignment of particles on the protein ring density rather than the membrane. Subtomogram refinement and sub-tilt refinement of translation and rotation, again with C12 symmetry applied, produced a final map at 9.9 Å resolution (Fig. 4C and fig. S6). After 10 iterations of symmetry-breaking refinement, a C1 map of the ring revealed asymmetric density within the central pore, shown in Fig. 4D. Refined ring particle orientations determined by subtomogram averaging were mapped to the originating tomograms using EMAN2 tool e2spt_*mapptclstotomo.py* (*66*, *67*). Segmentation of cellular features was performed using the convolutional neural net segmentation tool in EMAN2. Visualization and model docking were performed using UCSF ChimeraX and its built-in fit-in-map tool (*53*, *54*).

## Supplementary Materials

### This PDF file includes:

Figs. S1 to S8 Table S1 References
