# Supplementary material for: Molecular architecture of the Chikungunya virus replication complex
Source: Sci Adv. 2022 Nov 30;8(48):eadd2536. doi: 10.1126/sciadv.add2536 (PMC9710867; doi:10.1126/sciadv.add2536)
Supplement: Supplementary file 1 — Figs. S1 to S8 Table S1 References [file sciadv.add2536_sm.pdf]

Science Advances
AAAS

Supplementary Materials for

Molecular architecture of the Chikungunya virus replication complex

Yaw Bia Tan *et al.*

Corresponding author: David Chmielewski, david.chmielewski@stanford.edu; Jing Jin, jjin@vitalant.org; Dahai Luo, luodahai@ntu.edu.sg

Sci. Adv. **8**, eadd2536 (2022)
DOI: 10.1126/sciadv.add2536

The PDF file includes:

Figs. S1 to S8
Table S1
Legends for movies S1 and S2
References

Other Supplementary Material for this manuscript includes the following:

Movies S1 and S2

Figure S1 Cryo-EM analysis of nsP1+2+4 RC core complex. (A) cryoEM workflow in cryoSPARC (*51*) at C1 refinement symmetry. (B) GFSC resolutions of the map threshold at 0.143: masked versus unmasked. (C) map resolution overview and the overall geometrical position at the spherule. Scale bar blue-to-red: 2.5-4.5 Å (D) The nsP1+2+4 RC core complex (colored according to chain as Fig. 1) is superimposed to nsP1 dodecameric ring (PDB 7DOP here; colored green) (*10, 11*) at RMSD of 0.89 Å (between 416 Cα-pairs from each of nsP1), displayed here side-by-side on their top view. (E) The superimposition portrayed the confirmational difference at the hooking loop region (HL, dashed circle), focused at nsP1 chain A of nsP1+2+4 (purple) versus that of nsP1 (green). N- and C-terminal of nsP1 (chain A) are marked: the nsP1 C-terminal tails (aa 477-535 of nsP1 gene) remains unbuilt in the nsP1+2+4 due to missing map density, like the previously published nsP1 structures (*10, 11*).

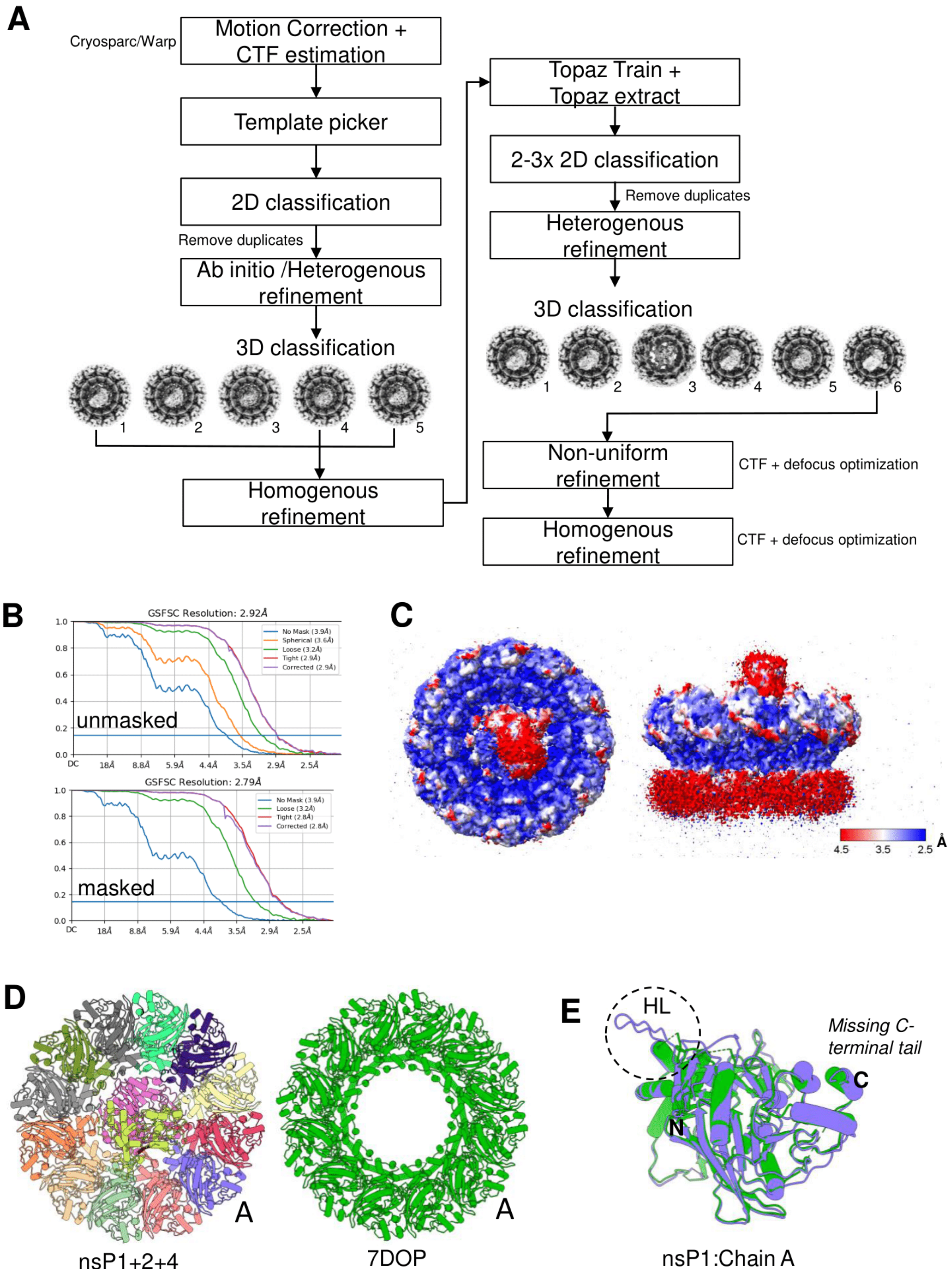
A
Cryosparc/Warp
Motion Correction + CTF estimation
Template picker
2D classification
Remove duplicates
Ab initio /Heterogenous refinement
3D classification
1
2
3
4
5
Homogenous refinement
Topaz Train + Topaz extract
2-3x 2D classification
Remove duplicates
Heterogenous refinement
3D classification
1
2
3
4
5
6
Non-uniform refinement
CTF + defocus optimization
Homogenous refinement
CTF + defocus optimization
B
GSFSC Resolution: 2.92Å
No Mask (3.9Å)
Spherical (3.6Å)
Loose (3.2Å)
Tight (2.9Å)
Corrected (2.9Å)
unmasked
GSFSC Resolution: 2.79Å
No Mask (3.9Å)
Loose (3.2Å)
Tight (2.8Å)
Corrected (2.8Å)
masked
C
4.5
3.5
2.5
Å
D
A
nsP1+2+4
A
7DOP
E
HL
Missing C-terminal tail
C
N
nsP1:Chain A

Figure S2. Map density fitting quality at the interaction interfaces and putative RNA channel within the RC core complex. The density map fitting (black mesh) on focused regions of (A) nsP2:nsP4 interface, (B) nsP2h-RNA interface, (C) nsP1:nsP4 interface at nsP1 chain L site, and (D) GDD in the nsP4 palm active site (also known as motif C and its first tyrosine residue (Y1) on nsP4 N-terminus tip. All parts are colored according to their respective protein: nsP4 in magenta, nsP2h NTD-Stalk-1B in tomato-yellow-cyan, RNA-bound in nsP2h in green, nsP1 chain L in red, nsP1 chain B in tomato, and nsP1 chain A in purple. (E) From the bottom view of the nsP1+2+4 complex, the putative channel of ~2.2nm diameter for replicated product RNA that may transport through the unbuilt density between nsP1 (chain A-B) and nsP4. (F) The ssRNA was placed as reference of size that is possible to transport through the putative channel.

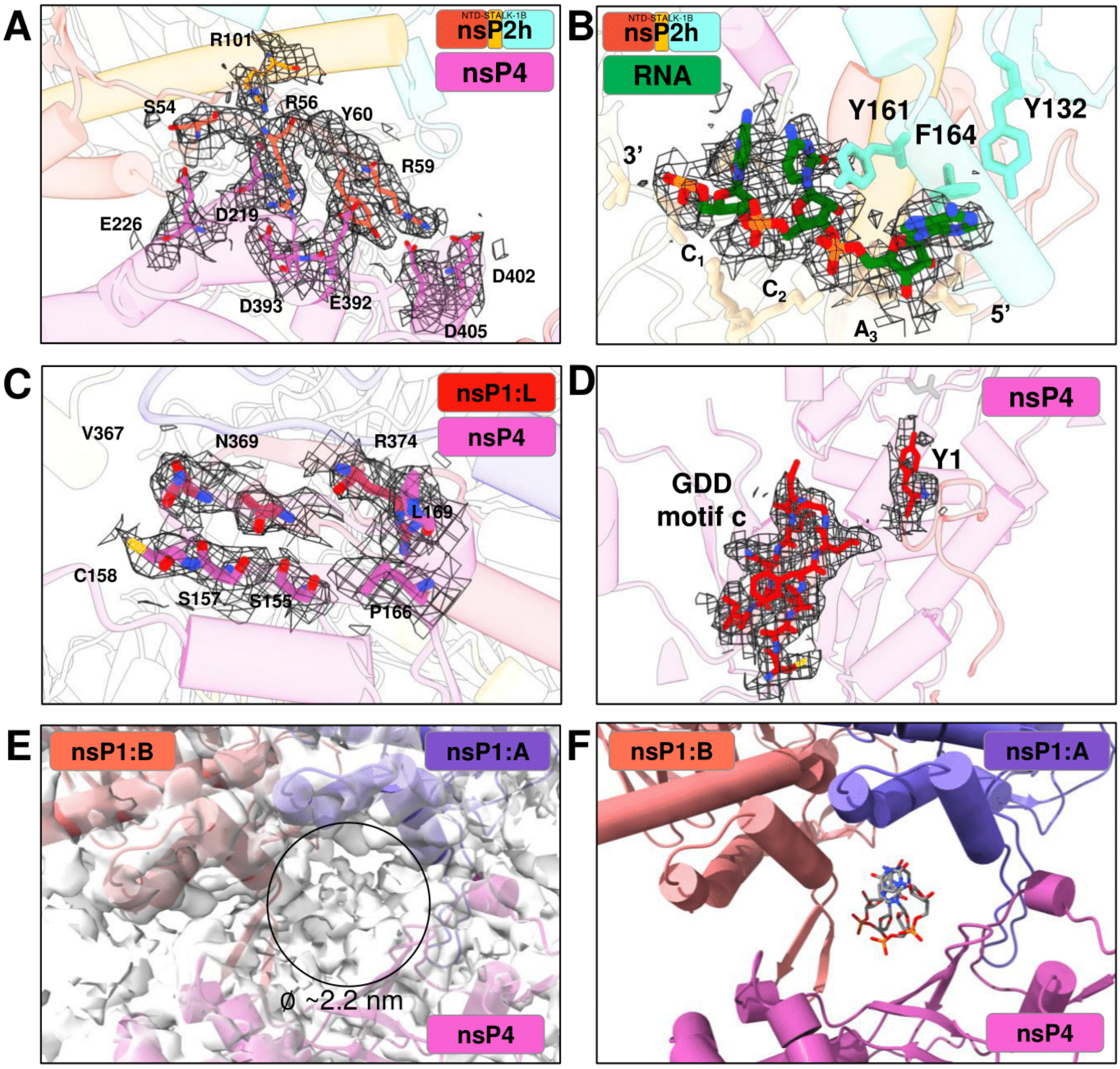

Figure S3. The structural interfaces in the RC and their sequence conservation. (A) The interaction of nsPs (nsP1: A-L; nsP4: X; nsP2-RNA: Y-Z) are presented here with their chain name in the form of network linking nodes and individual interface area. (B) The subdomain layouts of nsP2, nsP4 and nsP1 show their interacting interfaces via connections by black line. [Domain coloring sequence: nsP2h NTD-Stalk-1B (tomato-yellow-cyan), nsP4 NTD-Fingers-Palm-Thumb (red-green-gray-purple), nsP1 NTD-capping-MLA1-InnerRing-HL-MLA2-CTD (peach-light green-gray-pink-blue-gray-light yellow)] (C-F) The Weblogo-3 (*62*, *63*) style sequence conservations of the interacting residues are compared through multiple-sequence-alignment (MSA) among the alphaviruses on their nsPs. The interface residues of nsP2h:nsP4 are shown in boxes for nsP2h part in (C) while for nsP4 in (D). The interface residues which are highlighted here in boxes are for nsP4 in (E) and for nsP1 in (F). The sequences are colored based on charges (positive blue letter; negative red letter). *[Alphaviruses were used in MSA: chikungunya virus (CHIKV; NC_004162), o'nyong nyong virus (ONNV; AF079456.1), Semliki forest virus (SFV; NC_003215.1), Sindbis virus (SINV; NC_001547.1), Ross River virus (RRV; GQ433354.1), Mayaro virus (MAYV; NC_003417.1), Barmah forest virus (BFV; MN689034.1), Venezuelan equine encephalitis virus (VEEV; L01442.2); Eastern equine encephalitis virus (EEEV; EF151502.1), Western equine encephalitis virus (WEEV; MN477208.1); Eilat virus (EILV; NC_018615.1), and Getah virus (GETV; NC_006558.1).]*

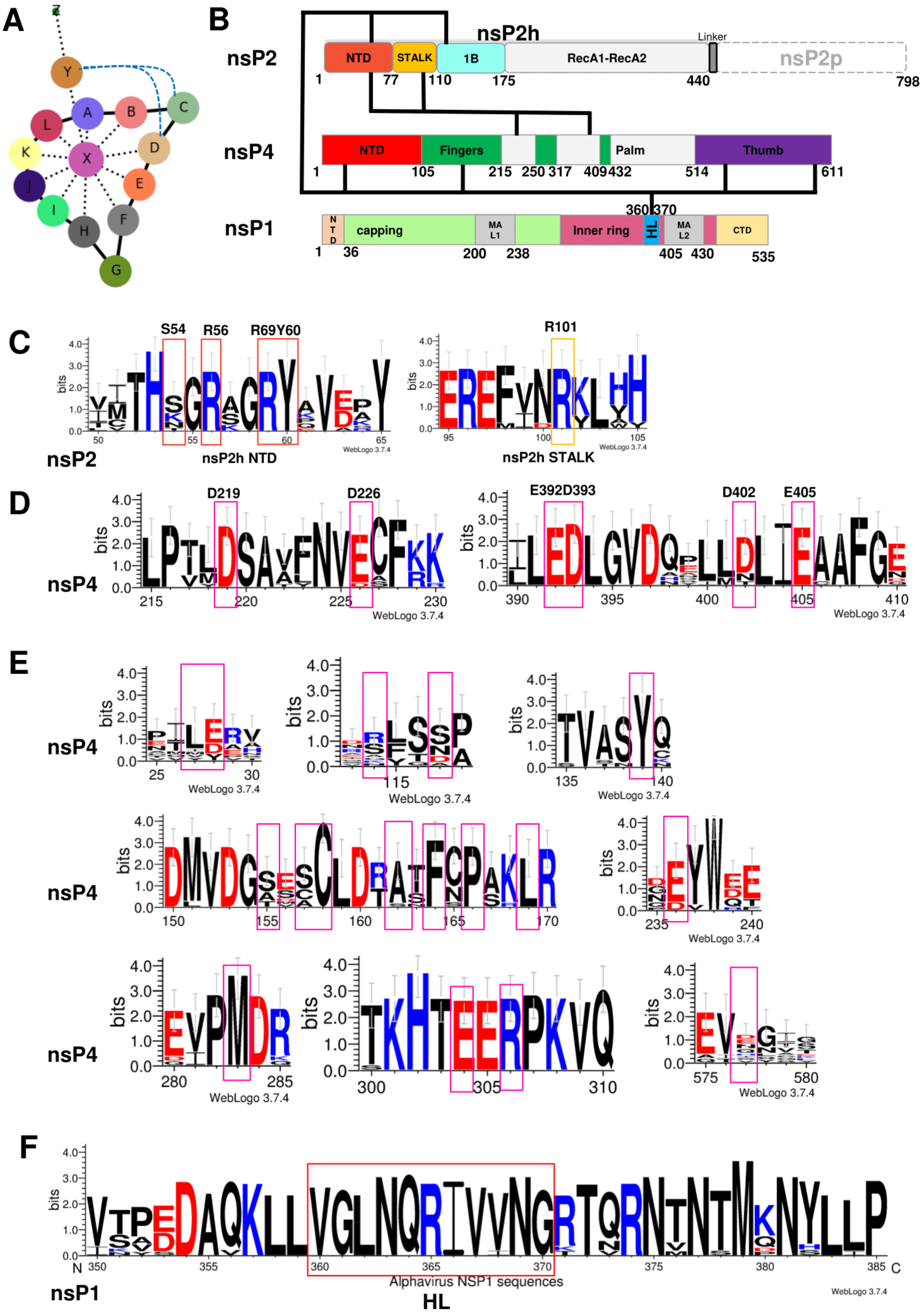
A
B
nsP2
nsP2h
NTD
STALK
1B
RecA1-RecA2
Linker
nsP2p
1
77
110
175
440
798
nsP4
NTD
Fingers
Palm
Thumb
1
105
215
250
317
409
432
514
611
nsP1
NTD
capping
MA L1
Inner ring
HL
MA L2
CTD
360
370
1
36
200
238
405
430
535
C
S54
R56
R69Y60
R101
nsP2
nsP2h NTD
nsP2h STALK
D
D219
D226
E392D393
D402
E405
nsP4
E
nsP4
nsP4
nsP4
F
Alphavirus NSP1 sequences
nsP1
HL

Figure S4. Comparison between cryoEM structure nsP4 from ONNV and homologous crystal structures of RdRp from alphavirus nsP4, *Picornaviridae* and *Flaviviridae*. (A) the cryoEM structure of nsP4 from ONNV was colored according to the subdomain (NTD: red; Index finger: green; Middle, ring, and pinky fingers: orange; Thumb: purple). (B) the crystal structures of the elongation complex from *Picornaviridae*: enterovirus-71 (EV71) RdRp with its RNA substrate (PDB 6KWQ) (RNA colored blue) (*33*). (C-D) The crystal structrures of alphavirus nsP4 homologs: RRV and SINV (PDB 7F0S and 7VB4) (*17*). (E) The crystal structure of another homolog from *Flaviviridae*: classical swine fever virus RdRp (CSFV; PDB 5Y6R) (*68*). ONNV nsP4 in (A) has RMSD of 1.0-1.2 Å (194 Cα-pairs aligned in aa215-575) at RdRp core domain when superimposed to nsP4 from RRV and SINV (*17*). The rearrangement of the index finger (black dotted arrow) is shown in (D) to form the folding in (A). When compared to EV71 RdRp and CSFV RdRp, they have respective RMSD of 1.3 Å and 1.26 Å (Cα-pairs aligned) at the RdRp core domain. *[RdRp coloring for (B-D): green Fingers, gray Palm and purple Thumb]*

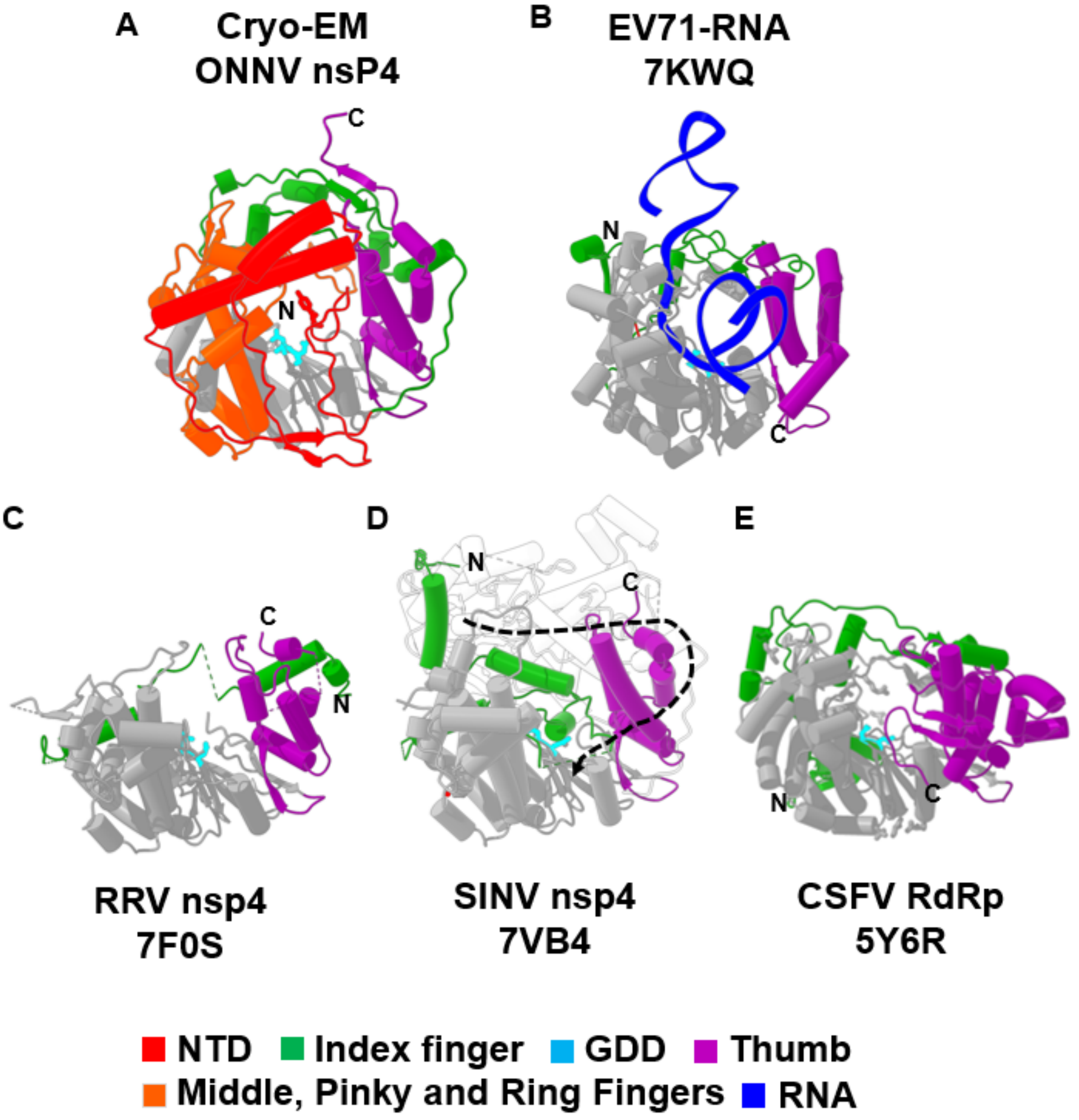

Figure S5. Subtomogram average map analysis of RC and non-replicative nsP complex. (A) RC density map shown as transparent surface with nsP1+4 atomic model (rainbow; nsP2 excluded) rigid fit into density. Single nsP1 subunit is computationally extracted (dark grey) and backbone of nsP1 subunit model is displayed (rainbow). (B) FSC curves of the masked and unmasked maps. (C) RC map colored by local resolution. (D) Non-replicative nsP ring density map displayed as transparent surface with nsP1+4 atomic model rigid fit into density, with single nsP1 subunit displayed. (E) Gold-standard FSC curve of masked and unmasked map with (F) density map colored by local resolution.

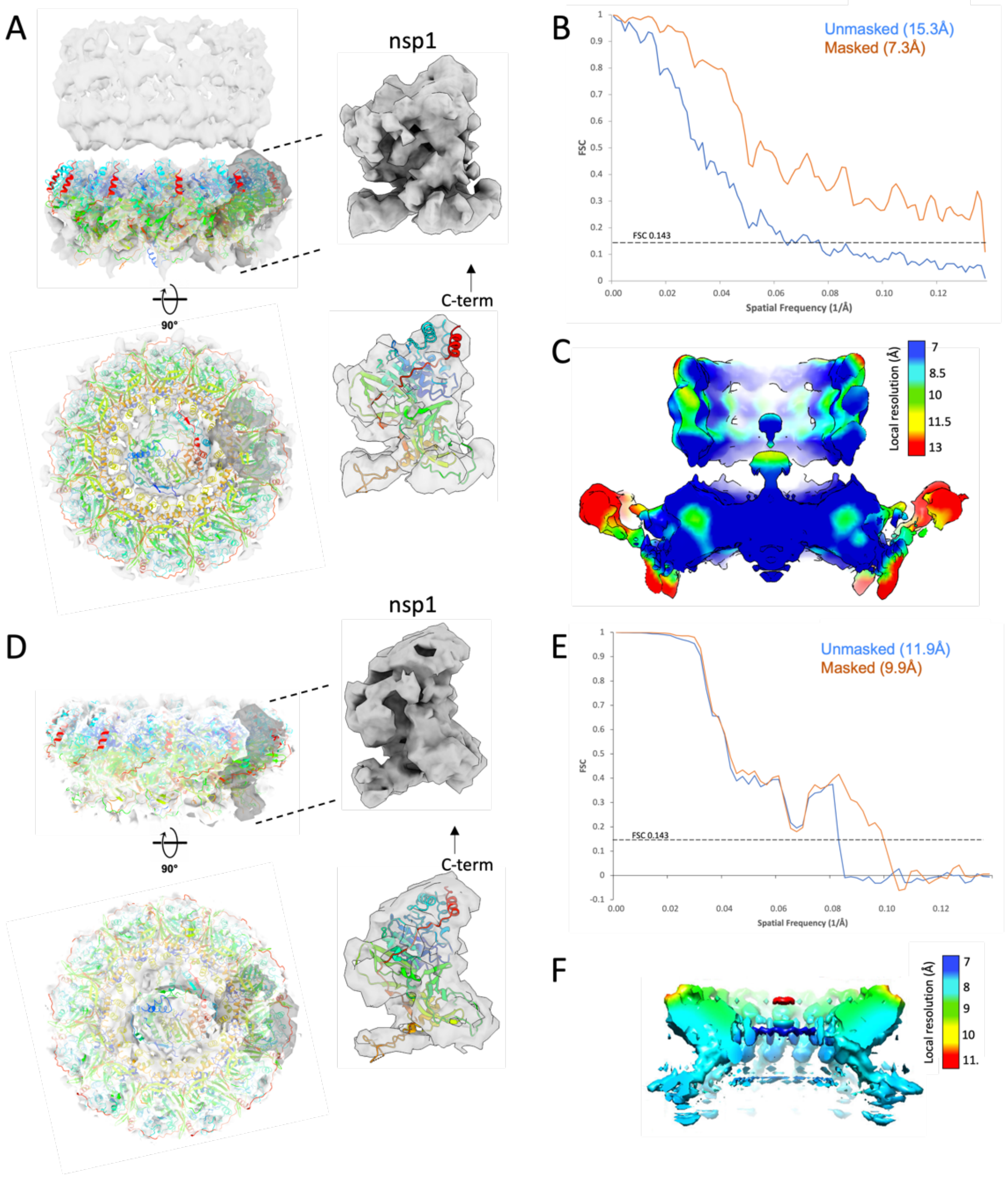

Figure S6. Topology of the (+) RNA virus replication complexes. Left: MHV nsp3 (gray) pore at the neck of the double-membrane vesicles (DMVs) derived from ER membrane (*19*). **Middle:** CHIKV replication complex at the neck of the spherule derived from the plasma membrane at C12 (cyan) and C1 (pink) symmetries. **Right:** FHV protein A crown (yellow) at the neck of the viral spherule derived from the mitochondrial outer membrane (*18*).

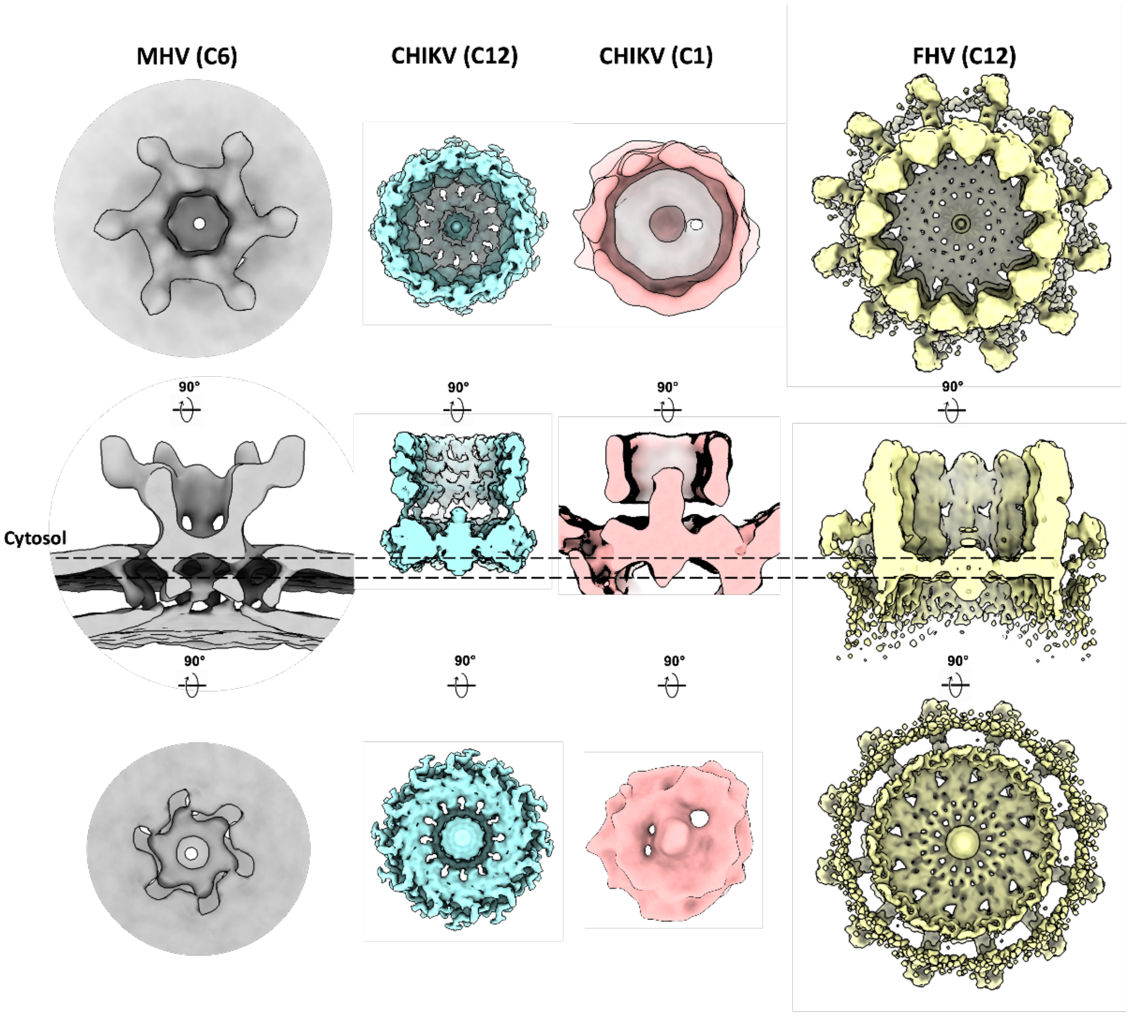

Figure S7. Non-replicative nsP complexes on the plasma membrane. (A) Low-mag montage of the cell periphery reveals numerous cell extensions (red arrows) emanating outward from the cell body. Scale bar 2 micron. (B) Slice image of a tomogram reconstruction displaying a representative thin cell extension. Zoom-in slice views of different membrane surfaces containing nsP complexes (white arrows). (C) Asymmetric 3D class averages of nsP complexes reveal presence of nsP1+4 in central pore of nsP1 ring in each class.

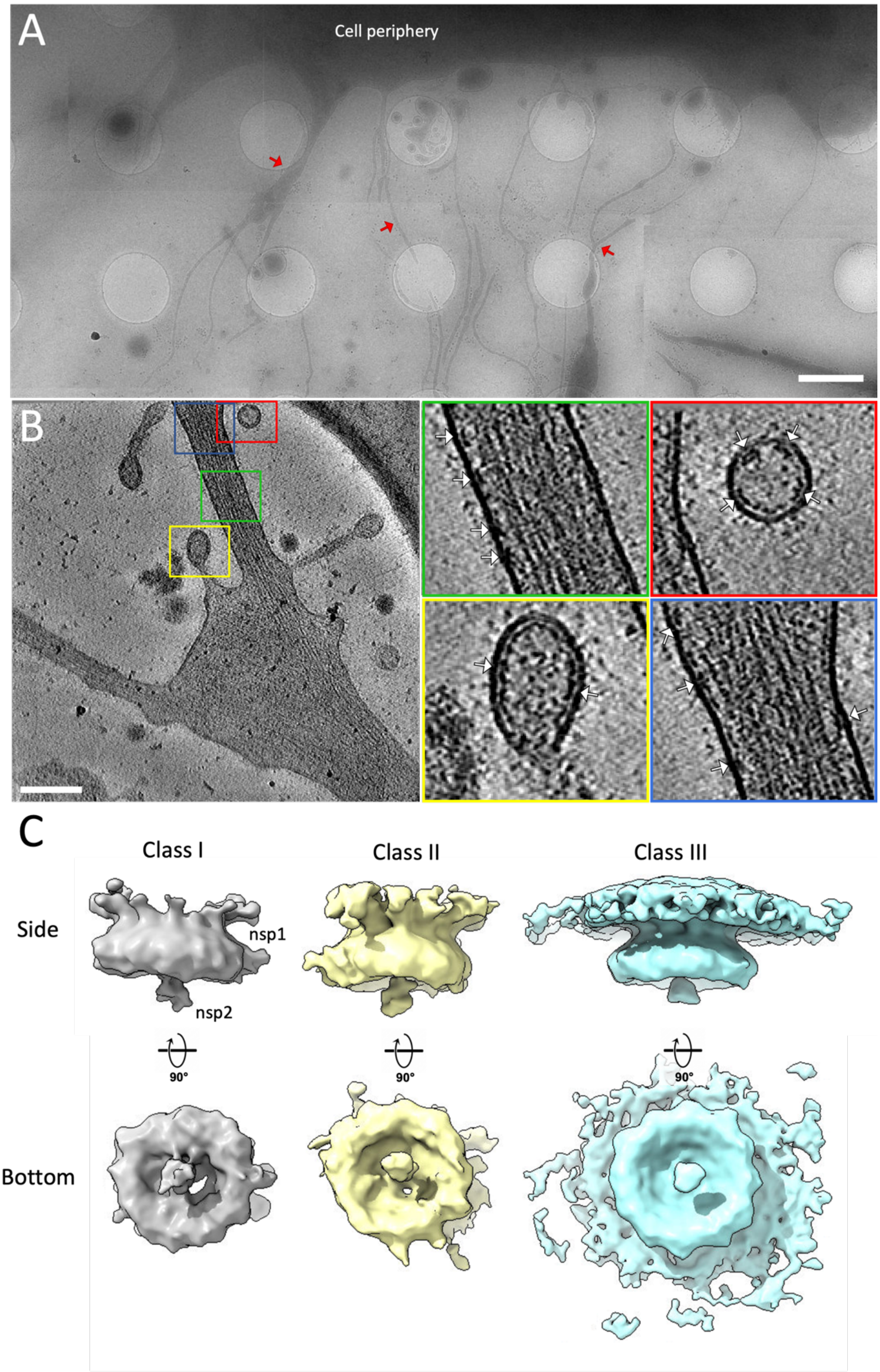

Figure S8. Membrane association of the active RC via the outer surface of the nsP1 ring. MA Loops 1 and 2 have been reported previously (*10, 11, 20*). The MA patches include mainly hydrophobic and positively charged residues 125-126, 269-270, 451-455, and 457-470 which collectively form a surface belt on the nsP1 upper ring.

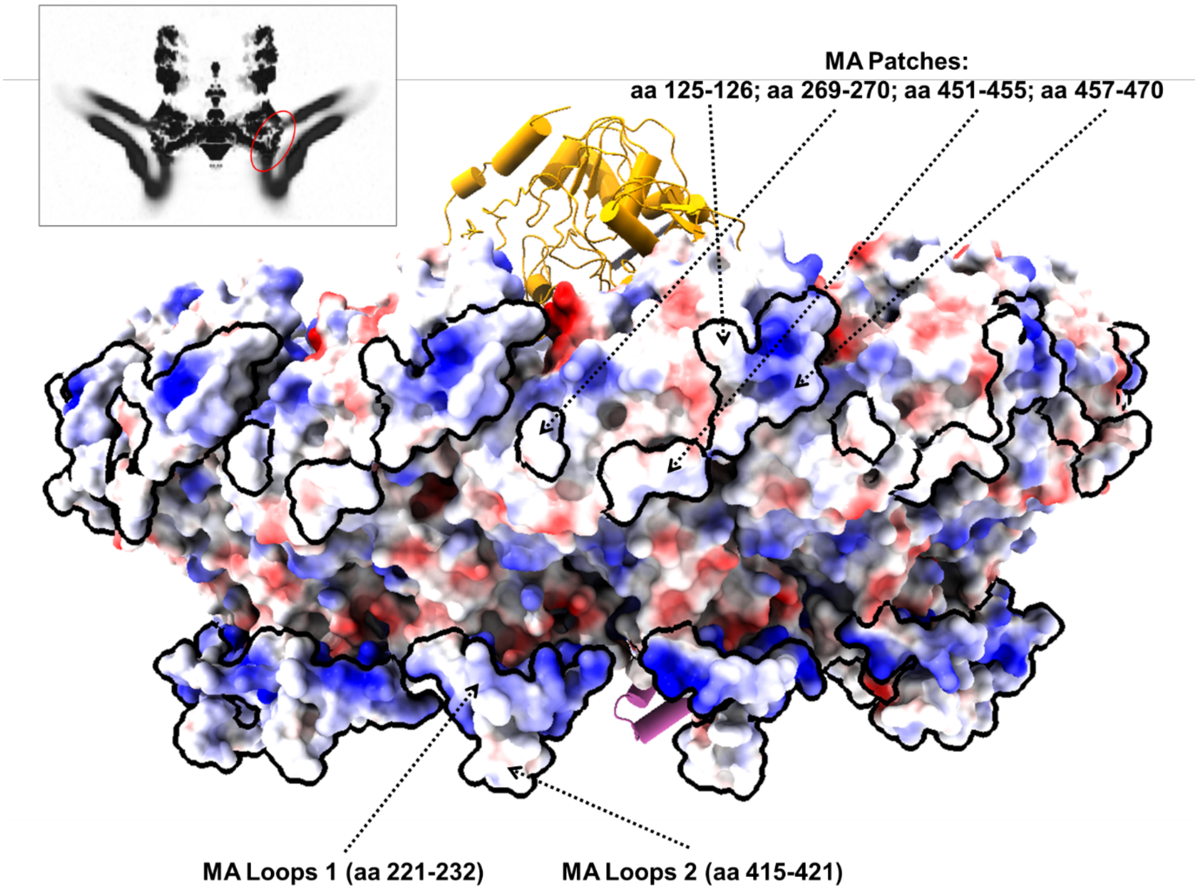

Table S1.

	nsP1+2+4<br>EMDB: 33591<br>PDB: 7Y38
**Data collection and processing**	
Detector	Gatan K2
Magnification	130,000 ×
Voltage (kV)	300
Electron exposure (e–/Å^2)	34
Defocus range (μm)	-0.5 ~-3.2
Pixel size (Å)	1.10
Symmetry imposed	C1
Initial particle images (no.)	898,911
Final particle images (no.)	132,510
Map resolution (Å)	2.8
FSC threshold micrographs	0.143
Map resolution range (Å)	2.4-6.2
**Refinement**	
Initial model used (PDB code)	7DOP (*10*)
Model resolution (Å)	2.8
FSC threshold	0.143-0.5
Model resolution range (Å)	2.8~3.3
Map sharpening B-factor (Å^2)	~63
**Model composition**	
Non-hydrogen atoms	53,139
Protein residues	Protein: 6704;
Nucleotide	3
Water	0
Ligands	ZN: 12<br>GTP: 12<br>ATP: 12
B factors (Å^2)	62.8
Protein	24.37/215.98/81.44
Nucleotide	215.58/231.42/224.21
Water	---
Ligand	56.25/126.14/65.90
**R.m.s. deviations**	
Bonds length (Å)	0.004
Bonds Angle (˚)	0.66
**Validation**	
MolProbity score	1.59
Clashscore	3.83
Average Q-score (*60*) at 2.8 Å	Protein: 0.57<br>Ligand: 0.66<br>Nucleotide: 0.31
Rotamer outliers (%)	0.05
**Ramachandran plot**	
Favored (%)	93.72

Allowed (%)	6.15
Disallowed (%)	0.14

Movie S1. (separate file)
CryoET tilt series of the CHIKV infected cell periphery shows the CHIKV RNA replication spherules with colored cellular features in 3D, corresponding to Fig. 3.

Movie S2. (separate file)
CryoET tilt series of the CHIKV infected cell periphery depicts a filopodia-like membrane protrusion structure extended from the plasma membrane, with 3D cellular features colored, corresponding to Fig. 4.

REFERENCES AND NOTES

1. P. Gasque, M. C. Bandjee, M. M. Reyes, D. Viasus, Chikungunya pathogenesis: From the clinics to the bench. *J. Infect. Dis.* **214**, S446–S448 (2016).

2. I. C. Albulescu, A. Tas, F. E. M. Scholte, E. J. Snijder, M. J. van Hemert, An in vitro assay to study chikungunya virus RNA synthesis and the mode of action of inhibitors. *J. Gen. Virol.* **95**, 2683–2692 (2014).

3. M. K. Pietila, M. J. van Hemert, T. Ahola, Purification of highly active alphavirus replication complexes demonstrates altered fractionation of multiple cellular membranes. *J. Virol.* **92**, e01852-17 (2018).

4. M. K. Pietilä, K. Hellström, T. Ahola, Alphavirus polymerase and RNA replication. *Virus Res.* **234**, 44–57 (2017).

5. I. Romero-Brey, R. Bartenschlager, Membranous replication factories induced by plus-strand RNA viruses. *Viruses* **6**, 2826–2857 (2014).

6. I. Romero-Brey, A. Merz, A. Chiramel, J. Y. Lee, P. Chlanda, U. Haselman, R. Santarella-Mellwig, A. Habermann, S. Hoppe, S. Kallis, P. Walther, C. Antony, J. Krijnse-Locker, R. Bartenschlager, Three-dimensional architecture and biogenesis of membrane structures associated with hepatitis C virus replication. *PLOS Pathog.* **8**, e1003056 (2012).

7. D. Paul, R. Bartenschlager, Flaviviridae replication organelles: Oh, what a tangled web we weave. *Annu. Rev. Virol.* **2**, 289–310 (2015).

8. D. Paul, R. Bartenschlager, Architecture and biogenesis of plus-strand RNA virus replication factories. *World J. Virol.* **2**, 32–48 (2013).

9. N. Unchwaniwala, H. Zhan, J. A. den Boon, P. Ahlquist, Cryo-electron microscopy of nodavirus RNA replication organelles illuminates positive-strand RNA virus genome replication. *Curr. Opin. Virol.* **51**, 74–79 (2021).

10. K. Zhang, Y.-S. Law, M. C. Y. Law, Y. B. Tan, M. Wirawan, D. Luo, Structural insights into viral RNA capping and plasma membrane targeting by Chikungunya virus nonstructural protein 1. *Cell Host Microbe* **29**, 757–764.e3 (2021).

11. K. Zhang, M. C. Y. Law, T. M. Nguyen, Y. B. Tan, M. Wirawan, Y.-S. Law, L. S. Jeong, D. Luo, Molecular basis of specific viral RNA recognition and 5′-end capping by the Chikungunya virus nsP1. *Cell Rep.* **40**, 111133 **40**, (2022).

12. Y.-S. Law, A. Utt, Y. B. Tan, J. Zheng, S. Wang, M. W. Chen, P. R. Griffin, A. Merits, D. Luo, Structural insights into RNA recognition by the Chikungunya virus nsP2 helicase. *Proc. Natl. Acad. Sci. U.S.A.* **116**, 9558–9567 (2019).

13. Y.-S. Law, S. Wang, Y. B. Tan, O. Shih, A. Utt, W. Y. Goh, B.-J. Lian, M. W. Chen, U.-S. Jeng, A. Merits, D. Luo, Interdomain flexibility of chikungunya virus nsP2 helicase-protease differentially influences viral rna replication and infectivity. *J. Virol.* **95**, e01470-20 (2021).

14. H. Malet, B. Coutard, S. Jamal, H. Dutartre, N. Papageorgiou, M. Neuvonen, T. Ahola, N. Forrester, E. A. Gould, D. Lafitte, F. Ferron, J. Lescar, A. E. Gorbalenya, X. de Lamballerie, B. Canard, The crystal structures of chikungunya and Venezuelan equine encephalitis virus nsP3 macro domains define a conserved adenosine binding pocket. *J. Virol.* **83**, 6534–6545 (2009).

15. G. Nowee, J. W. Bakker, C. Geertsema, V. I. D. Ros, G. P. Göertz, J. J. Fros, G. P. Pijlman, A tale of 20 alphaviruses; inter-species diversity and conserved interactions between viral non-structural protein 3 and stress granule proteins. *Front. Cell Dev. Biol.* **9**, 625711 (2021).

16. M. W. Chen, Y. B. Tan, J. Zheng, Y. Zhao, B. T. Lim, T. Cornvik, J. Lescar, L. F. P. Ng, D. Luo, Chikungunya virus nsP4 RNA-dependent RNA polymerase core domain displays detergent-sensitive primer extension and terminal adenylyltransferase activities. *Antiviral Res.* **143**, 38–47 (2017).

17. Y. B. Tan, L. S. Lello, X. Liu, Y. S. Law, C. Kang, J. Lescar, J. Zheng, A. Merits, D. Luo, Crystal structures of alphavirus nonstructural protein 4 (nsP4) reveal an intrinsically dynamic RNA-dependent RNA polymerase fold. *Nucleic Acids Res.* **50**, 1000–1016 (2022).

18. N. Unchwaniwala, H. Zhan, J. Pennington, M. Horswill, J. A. den Boon, P. Ahlquist, Subdomain cryo-EM structure of nodaviral replication protein A crown complex provides mechanistic insights into RNA genome replication. *Proc. Natl. Acad. Sci. U.S.A.* **117**, 18680–18691 (2020).

19. G. Wolff, R. W. A. L. Limpens, J. C. Zevenhoven-Dobbe, U. Laugks, S. Zheng, A. W. M. de Jong, R. I. Koning, D. A. Agard, K. Grünewald, A. J. Koster, E. J. Snijder, M. Bárcena, A molecular pore spans the double membrane of the coronavirus replication organelle. *Science* **369**, 1395–1398 (2020).

20. R. Jones, G. Bragagnolo, R. Arranz, J. Reguera, Capping pores of alphavirus nsP1 gate membranous viral replication factories. *Nature* **589**, 615–619 (2021).

21. K. D. Saxton-Shaw, J. P. Ledermann, E. M. Borland, J. L. Stovall, E. C. Mossel, A. J. Singh, J. Wilusz, A. M. Powers, O'nyong nyong virus molecular determinants of unique vector specificity reside in non-structural protein 3. *PLoS Negl. Trop. Dis.* **7**, e1931 (2013).

22. L. S. Lello, K. Bartholomeeusen, S. Wang, S. Coppens, R. Fragkoudis, L. Alphey, K. K. Ariën, A. Merits, A. Utt, nsP4 is a major determinant of alphavirus replicase activity and template selectivity. *J. Virol.* **95**, e0035521 (2021).

23. I. Frolov, E. I. Frolova, Molecular virology of chikungunya virus. *Curr. Top. Microbiol. Immunol.* **435**, 1–31 (2022).

24. T. Ahola, G. McInerney, A. Merits, Alphavirus RNA replication in vertebrate cells. *Adv. Virus Res.* **111**, 111–156 (2021).

25. B. Thaa, R. Biasiotto, K. Eng, M. Neuvonen, B. Götte, L. Rheinemann, M. Mutso, A. Utt, F. Varghese, G. Balistreri, A. Merits, T. Ahola, G. M. McInerney, Differential phosphatidylinositol-3-kinase-akt-mTOR activation by semliki forest and chikungunya viruses is dependent on nsP3 and connected to replication complex internalization. *J. Virol.* **89**, 11420–11437 (2015).

26. T. Ahola, G. McInerney, A. Merits, in *Advances in Virus Research*, M. Kielian, T. C. Mettenleiter, M. J. Roossinck, Eds. (Academic Press, 2021), vol. 111, pp. 111–156.

27. E. I. Frolova, R. Gorchakov, L. Pereboeva, S. Atasheva, I. Frolov, Functional sindbis virus replicative complexes are formed at the plasma membrane. *J. Virol.* **84**, 11679–11695 (2010).

28. B. Götte, L. Liu, G. M. McInerney, The enigmatic alphavirus non-structural protein 3 (nsP3) revealing its secrets at last. *Viruses* **10**, 105 (2018).

29. P. Laakkonen, T. Ahola, L. Kääriäinen, The effects of palmitoylation on membrane association of semliki forest virus RNA capping enzyme. *J. Biol. Chem.* **271**, 28567–28571 (1996).

30. P. Laakkonen, P. Auvinen, P. Kujala, L. Kääriäinen, Alphavirus replicase protein NSP1 induces filopodia and rearrangement of actin filaments. *J. Virol.* **72**, 10265–10269 (1998).

31. Y. Furuta, T. Komeno, T. Nakamura, Favipiravir (T-705), a broad spectrum inhibitor of viral RNA polymerase. *Proc. Jpn. Acad. Ser. B Phys. Biol. Sci.* **93**, 449–463 (2017).

32. F. Picarazzi, I. Vicenti, F. Saladini, M. Zazzi, M. Mori, Targeting the RdRp of emerging RNA Viruses: The structure-based drug design challenge. *Molecules* **25**, 5695 (2020).

33. W. Shi, H.-Q. Ye, C.-L. Deng, R. Li, B. Zhang, P. Gong, A nucleobase-binding pocket in a viral RNA-dependent RNA polymerase contributes to elongation complex stability. *Nucleic Acids Res.* **48**, 1392–1405 (2020).

34. J. Wu, W. Liu, P. Gong, A structural overview of RNA-dependent RNA polymerases from the flaviviridae family. *Int. J. Mol. Sci.* **16**, 12943–12957 (2015).

35. H. S. Hillen, G. Kokic, L. Farnung, C. Dienemann, D. Tegunov, P. Cramer, Structure of replicating SARS-CoV-2 polymerase. *Nature* **584**, 154–156 (2020).

36. Q. Peng, R. Peng, B. Yuan, J. Zhao, M. Wang, X. Wang, Q. Wang, Y. Sun, Z. Fan, J. Qi, G. F. Gao, Y. Shi, Structural and biochemical characterization of the nsp12-nsp7-nsp8 core polymerase complex from SARS-CoV-2. *Cell Rep.* **31**, 107774 (2020).

37. Q. Wang, J. Wu, H. Wang, Y. Gao, Q. Liu, A. Mu, W. Ji, L. Yan, Y. Zhu, C. Zhu, X. Fang, X. Yang, Y. Huang, H. Gao, F. Liu, J. Ge, Q. Sun, X. Yang, W. Xu, Z. Liu, H. Yang, Z. Lou, B. Jiang, L. W.

Guddat, P. Gong, Z. Rao, Structural basis for RNA replication by the SARS-CoV-2 polymerase. *Cell* **182**, 417–428.e13 (2020).

38. T. L. Yap, T. Xu, Y.-L. Chen, H. Malet, M.-P. Egloff, B. Canard, S. G. Vasudevan, J. Lescar, Crystal structure of the dengue virus RNA-dependent RNA polymerase catalytic domain at 1.85-angstrom resolution. *J. Virol.* **81**, 4753–4765 (2007).

39. S. Venkataraman, B. V. L. S. Prasad, R. Selvarajan, RNA dependent RNA polymerases: Insights from structure, function and evolution. *Viruses* **10**, 76 (2018).

40. T. C. Appleby, J. K. Perry, E. Murakami, O. Barauskas, J. Feng, A. Cho, D. Fox III, D. R. Wetmore, M. E. McGrath, A. S. Ray, M. J. Sofia, S. Swaminathan, T. E. Edwards, Structural basis for RNA replication by the hepatitis C virus polymerase. *Science* **347**, 771–775 (2015).

41. M. E. Fairman-Williams, U.-P. Guenther, E. Jankowsky, SF1 and SF2 helicases: Family matters. *Curr. Opin. Struct. Biol.* **20**, 313–324 (2010).

42. P. Spuul, A. Salonen, A. Merits, E. Jokitalo, L. Kääriäinen, T. Ahola, Role of the amphipathic peptide of semliki forest virus replicase protein nsP1 in membrane association and virus replication. *J. Virol.* **81**, 872–883 (2007).

43. J. Peränen, P. Laakkonen, M. Hyvönen, L. Kääriäinen, The alphavirus replicase protein nsP1 is membrane-associated and has affinity to endocytic organelles. *Virology* **208**, 610–620 (1995).

44. T. Ahola, P. Kujala, M. Tuittila, T. Blom, P. Laakkonen, A. Hinkkanen, P. Auvinen, Effects of palmitoylation of replicase protein nsP1 on alphavirus infection. *J. Virol.* **74**, 6725–6733 (2000).

45. K. J. Ertel, D. Benefield, D. Castaño-Diez, J. G. Pennington, M. Horswill, J. A. den Boon, M. S. Otegui, P. Ahlquist, Cryo-electron tomography reveals novel features of a viral RNA replication compartment. *eLife* **6**, e25940 (2017).

46. I. Akhrymuk, S. V. Kulemzin, E. I. Frolova, Evasion of the innate immune response: The old world alphavirus nsP2 protein induces rapid degradation of Rpb1, a catalytic subunit of RNA polymerase II. *J. Virol.* **86**, 7180–7191 (2012).

47. B. D. Carey, A. Bakovic, V. Callahan, A. Narayanan, K. Kehn-Hall, New World alphavirus protein interactomes from a therapeutic perspective. *Antiviral Res.* **163**, 125–139 (2019).

48. N. Bhalla, C. Sun, L. K. Metthew Lam, C. L. Gardner, K. D. Ryman, W. B. Klimstra, Host translation shutoff mediated by non-structural protein 2 is a critical factor in the antiviral state resistance of Venezuelan equine encephalitis virus. *Virology* **496**, 147–165 (2016).

49. Y. Han, X. Fan, H. Wang, F. Zhao, C. G. Tully, J. Kong, N. Yao, N. Yan, High-yield monolayer graphene grids for near-atomic resolution cryoelectron microscopy. *Proc. Natl. Acad. Sci. U.S.A.* **117**, 1009–1014 (2020).

50. D. Tegunov, P. Cramer, Real-time cryo-electron microscopy data preprocessing with Warp. *Nat. Methods* **16**, 1146–1152 (2019).

51. A. Punjani, J. L. Rubinstein, D. J. Fleet, M. A. Brubaker, cryoSPARC: Algorithms for rapid unsupervised cryo-EM structure determination. *Nat. Methods* **14**, 290–296 (2017).

52. M. Wolf, D. J. DeRosier, N. Grigorieff, Ewald sphere correction for single-particle electron microscopy. *Ultramicroscopy* **106**, 376–382 (2006).

53. T. D. Goddard, C. C. Huang, E. C. Meng, E. F. Pettersen, G. S. Couch, J. H. Morris, T. E. Ferrin, UCSF ChimeraX: Meeting modern challenges in visualization and analysis. *Protein Sci.* **27**, 14–25 (2018).

54. E. F. Pettersen, T. D. Goddard, C. C. Huang, E. C. Meng, G. S. Couch, T. I. Croll, J. H. Morris, T. E. Ferrin, UCSF ChimeraX: Structure visualization for researchers, educators, and developers. *Protein Sci.* **30**, 70–82 (2021).

55. J. Pfab, N. M. Phan, D. Si, DeepTracer for fast de novo cryo-EM protein structure modeling and special studies on CoV-related complexes. *Proc. Natl. Acad. Sci. U.S.A.* **118**, e2017525118 (2021).

56. R. Evans, M. O’Neill, A. Pritzel, N. Antropova, A. Senior, T. Green, Augustin Žídek, R. Bates, S. Blackwell, J. Yim, O. Ronneberger, S. Bodenstein, Michal, Zielinski, A. Bridgland, A. Potapenko, A. Cowie, K. Tunyasuvunakool, R. Jain, Ellen, Clancy, P. Kohli, J. Jumper, D. Hassabis, Protein complex

prediction with AlphaFold-Multimer. bioRxiv 463934 [**Preprint**]. 10 March 2022. https://doi.org/10.1101/2021.10.04.463034.

57. M. Mirdita, K. Schütze, Y. Moriwaki, L. Heo, S. Ovchinnikov, M. Steinegger, ColabFold—Making protein folding accessible to all. bioRxiv 456425 [**Preprint**]. 15 August 2021. https://doi.org/10.1101/2021.08.15.456425.

58. P. Emsley, B. Lohkamp, W. G. Scott, K. Cowtan, Features and development of Coot. *Acta Crystallogr. D Biol. Crystallogr.* **66**, 486–501 (2010).

59. D. Liebschner, P. V. Afonine, M. L. Baker, G. Bunkóczi, V. B. Chen, T. I. Croll, B. Hintze, L. W. Hung, S. Jain, A. J. McCoy, N. W. Moriarty, R. D. Oeffner, B. K. Poon, M. G. Prisant, R. J. Read, J. S. Richardson, D. C. Richardson, M. D. Sammito, O. V. Sobolev, D. H. Stockwell, T. C. Terwilliger, A. G. Urzhumtsev, L. L. Videau, C. J. Williams, P. D. Adams, Macromolecular structure determination using X-rays, neutrons and electrons: Recent developments in Phenix. *Acta Crystallogr. Sec. D Struct. Biol.* **75**, 861–877 (2019).

60. G. Pintilie, K. Zhang, Z. Su, S. Li, M. F. Schmid, W. Chiu, Measurement of atom resolvability in cryo-EM maps with Q-scores. *Nat. Methods* **17**, 328–334 (2020).

61. F. Madeira, Y. . Park, J. Lee, N. Buso, T. Gur, N. Madhusoodanan, P. Basutkar, A. R. N. Tivey, S. C. Potter, R. D. Finn, R. Lopez, The EMBL-EBI search and sequence analysis tools APIs in 2019. *Nucleic Acids Res.* **47**, W636–W641 (2019).

62. G. E. Crooks, G. Hon, J.-M. Chandonia, S. E. Brenner, WebLogo: A sequence logo generator. *Genome Res.* **14**, 1188–1190 (2004).

63. T. D. Schneider, R. M. Stephens, Sequence logos: A new way to display consensus sequences. *Nucleic Acids Res.* **18**, 6097–6100 (1990).

64. D. N. Mastronarde, Automated electron microscope tomography using robust prediction of specimen movements. *J. Struct. Biol.* **152**, 36–51 (2005).

65. S. Q. Zheng, E. Palovcak, J. P. Armache, K. A. Verba, Y. Cheng, D. A. Agard, MotionCor2: Anisotropic correction of beam-induced motion for improved cryo-electron microscopy. *Nat. Methods* **14**, 331–332 (2017).

66. J. G. Galaz-Montoya, J. Flanagan, M. F. Schmid, S. J. Ludtke, Single particle tomography in EMAN2. *J. Struct. Biol.* **190**, 279–290 (2015).

67. G. Tang, L. Peng, P. R. Baldwin, D. S. Mann, W. Jiang, I. Rees, S. J. Ludtke, EMAN2: An extensible image processing suite for electron microscopy. *J. Struct. Biol.* **157**, 38–46 (2007).

68. W. Li, B. Wu, W. A. Soca, L. An, T. Gallagher, Crystal structure of classical swine fever virus NS5B reveals a novel N-terminal domain. *J. Virol.* **92**, e00324-18 (2018).
